# Obsidian, Waste Ceramic Powder, and Recycled Concrete Powder as Alternative Aggregates in Hydroxypropyl Methylcellulose-Stabilized Foamed Concrete: Mechanical, Thermal, and Durability Performance

**DOI:** 10.3390/polym18182192

**Published:** 2026-09-08

**Authors:** Kenan Mert Oksuz, Talip Çakmak, İlker Ustabaş, Zafer Kurt

**Affiliations:** 1 Department of Civil Engineering, Recep Tayyip Erdogan University, Fener, Rize 53100, Türkiye; kenanmert_oksuz23@erdogan.edu.tr (K.M.O.); zafer.kurt@erdogan.edu.tr (Z.K.); 2 Department of Construction Technology, Dokuz Eylül University, Buca, Izmir 35390, Türkiye; talip.cakmak@deu.edu.tr

**Keywords:** foamed concrete, obsidian, sustainability, compressive strength, durability, thermal conductivity, microstructure

## Abstract

The substitution of conventional materials with alternative resources is a significant approach for enhancing the engineering performance and sustainability of foamed concrete (FC). While supplementary cementitious materials, volcanic materials, and waste-derived materials have been extensively investigated, the use of obsidian as an alternative aggregate in FC systems remains largely unexplored, and the combined, systematic comparative use of obsidian, waste ceramic powder (WCP), and recycled concrete powder (RCP) within a unified experimental framework has not been previously investigated. This paper evaluates the use of obsidian, WCP, and RCP as alternative aggregates in hydroxypropyl methylcellulose (HPMC)-stabilized FC by replacing standard sand at 25%, 50%, and 100% levels. The thermal, durability and mechanical characteristics of the mixtures were assessed through density, compressive strength (CS), ultrasonic pulse velocity (UPV), water absorption (WA), elevated temperature resistance (200 °C, 400 °C, 600 °C and 800 °C), freeze–thaw performance, thermal conductivity (TC), and scanning electron microscopy coupled with energy-dispersive X-ray spectroscopy (SEM–EDS) and X-ray diffraction (XRD) analyses. The results showed that the 28-day CS increased from 0.545 MPa in the control mixture to a maximum value of 2.590 MPa in the obsidian-based FC. Moreover, WA decreased markedly from 117.7% to 46.9% in the obsidian-based FC. The UPV varied from 1355 to 1795 m/s due to the incorporation of RCP, WCP and obsidian at different replacement ratios in the mixture designs. The lowest TC of 0.08185 W/(m·K) was recorded in the obsidian-based FC at 50% substitution level. Under elevated-temperature exposure, the mixture with 100% obsidian replacement retained a compressive strength of 0.5936 MPa at 800 °C. To conclude, the use of obsidian, WCP and RCP as alternative aggregates in FC shows promising potential for the development of durable, thermally efficient, and sustainable lightweight construction materials.

## 1. Introduction

Concrete is one of the major composites most employed in modern structural systems and its sustainability is in the focus of current research due to the environmental impacts arising from raw material supply and production processes [1,2,3,4]. FC, offering features of particularly low density, acoustic and high thermal insulation performance, fire resistance, and workability, is considered a prominent material in building applications due to its potential to reduce energy consumption, lower labor requirements, and contribute to structural efficiency by reducing dead loads in load-bearing systems [5,6,7]. However, the elevated WA capacity of FC compared to ordinary concrete, and the capillary void network resulting from its porous structure, accelerate the transport of harmful ions, limiting its durability performance. The effects of parameters such as production method, binder and aggregate type, curing conditions, stabilizer use, and fiber reinforcement on mitigating this effect and improving long-term performance are extensively studied in the literature [8,9,10,11,12,13,14]. However, the effects of these approaches on performance differ depending on the form of material applied and the void distribution; the interaction between porosity and mechanical strength and WA behavior makes it difficult to obtain an optimized performance between low unit weight, mechanical strength and durability characteristics [15,16,17]. In this context, alternative aggregate sources, including various options such as recycled aggregates, industrial by-products and materials of different mineral origins, stand out as an important research area in relation to enhancing the characteristics of the properties of FC [18,19,20,21].

FC is at the center of systematic scientific research aimed at comprehensively understanding its mechanical performance, long-term durability, and microstructural properties [22,23,24,25,26]. FC is manufactured by integrating pre-formed foam into a cement-bonded slurry or by directly foaming the mixture and this structure is characterized by high porosity and low density [27,28,29]. This low density is due to the fine and homogeneous air voids created by foaming agents within the cement paste [30]. The foaming agents used in this context can be synthetic, protein-based, and plant-derived, and the type of agent plays a decisive role in foam stability and final material performance [31,32,33]. The fact that it can be produced using different types of binders, mineral admixtures, and alternative aggregate types of both natural and waste origin shows that FC has a flexible structure in terms of composition [34,35,36]. In this context, proportional or full substitution of traditional components with alternative materials in FC mixtures contributes to reducing material consumption while also offering significant environmental and economic advantages [37,38,39]. However, the lack of a standard mix design method and variations in foam stability, mix ratios and curing conditions directly affect the performance of this material and restrict its widespread use [40,41].

RCP, obtained from processed construction and demolition waste, is considered a fine secondary material derived from previously hydrated cementitious systems, where its residual clinker-related phases and irregular particle morphology provide a functional contribution to the cementitious matrix through micro-filler action and interfacial transition-zone modification mechanisms, and its incorporation offers a viable route for tailoring matrix densification and pore structure regulation [42,43,44]. WCP, generated from ceramic-based industrial and construction residues, is regarded as a finely divided aluminosilicate material characterized by a stable crystalline–amorphous hybrid structure and high silica–alumina content, where its primary function is associated with particle-packing optimization and microstructural refinement mechanisms, contributing to improved matrix continuity and controlled pore-architecture development [45,46,47,48]. Obsidian, a natural glass of volcanic origin, has been reported to exhibit pozzolanic potential in cement and alkali-activated binder-based composite systems due to its high silica content and amorphous structure [49,50,51,52]. For example, He et al. [53] evaluated the incorporation of waste-derived products in FC and reported that when Al-based residues and coral-derived sand were used in the roles of foam agents and filler materials respectively, the mechanical, acoustic and thermal characteristics of the material changed substantially, the CS decreased to the range of 1.26–1.59 MPa in mixtures where the foam content reached 15%, the TC declined by 52.5% relative to the control samples, reaching a lowest of 0.082 W/(m·K) and the most suitable results in terms of noise reduction performance were obtained at 10% foam content. Bayraktar et al. [54] assessed the use of recycled brick (BR) and tile (RT) fine-grained particles in place of limestone aggregate in FC and found that mechanical properties improved at a 10% substitution rate, while at higher rates (20% and 40%), strength decreased due to increased porosity, and density, WA and porosity values showed similar trends. Mixtures containing BR exhibited higher resistance to drying shrinkage, freeze–thaw and high temperature effects compared to RT, and the optimum substitution rates were generally at 10% (RT) and 20% (BR) levels. Zhang et al. [55] characterized the behavior of FC containing shale aggregate and, as a result of experiments carried out on foams at 60 and 80 kg/m^3^, stated that the use of shale aggregate created an improved void structure characterized by high sphericity (0.75–0.85) and retained a fine pore structure, and consequently, CS and flexural strength reached 26.5 MPa and 7.0 MPa correspondingly, mechanical performance was significantly increased compared to conventional sand-based mixtures, cement content was reduced from 1120.5 to 661.2 kg/m^3^, drying shrinkage was reduced by 52% and WA values were maintained at a low level. Siva et al. [56] assessed the integration of a large proportion of fly ash in FC with a rate of 0–100% instead of fine aggregate and found that the addition of fly ash prevented void coalescence, creating a more refined void structure and increasing CS, while sorptivity and drying shrinkage increased with increasing fly ash content, and also resulted in a lower foam volume requirement at constant density and reduced WA at foam volume levels of 30%. Liu et al. [57] observed the effect of grain size and gradation of perlite aggregate on FC and reported that the dry unit weight declined from 1130.6 kg/m^3^ to 972.5 kg/m^3^ and the WA increased from 17.5% to 23.0%, while the CS showed a decreasing trend. The highest CS was obtained at 0.5–1 mm grain size with 9.12 MPa, and the 30:40:30 ratio in the multiple grain distribution provided the second best performance with 8.06 MPa. Furthermore, the lowest TC was determined as 0.1194 W/(m·K), and the addition of perlite increased the TC while increasing the CS compared to the control samples. Gencel et al. [58] investigated the use of recycled fine concrete aggregate (RFA) as a replacement for natural sand in foam concrete at 0%, 25%, 50%, 75% and 100% ratios and reported that the increase in RFA content resulted in a slight increase in porosity and water absorption, a decrease in dynamic elastic modulus, ultrasonic pulse velocity and thermal conductivity. The dry unit weight decreased slightly with the increase in RFA content due to the lower specific gravity of RFA compared with natural sand, whereas foam concrete containing up to 50% RFA exhibited compressive strength comparable to the control mixture. Awoyera and Britto [59] investigated the use of pulverized ceramics as a replacement for river sand in foam concrete containing fly ash, and reported that mixtures with 100% ceramic aggregate achieved compressive strength close to that of the conventional river sand-based mixture, attributed to improved particle packing and filler effect. However, the workability of the ceramics-based mixtures was lower due to the high water absorption capacity of the ceramic aggregate. However, the existing literature, as highlighted by the studies mentioned above, has mostly focused on the utilization of single waste-derived or natural aggregate substitutes in FC systems in isolation, while a systematic comparative assessment of multiple alternative aggregate types within a single common experimental framework is still missing. In particular, RCP and WCP have been investigated individually as sand replacement in FC, while the behavior of obsidian remains largely unexplored in FC systems. Therefore, this study presents the first systematic investigation that comparatively evaluates obsidian, RCP, and WCP as alternative aggregates within a unified experimental framework in FC, with the objective of elucidating their individual and relative effects on mechanical, physical strength, and microstructural performance and thereby providing a new perspective on sustainable and high-performance FC design strategies.

These materials were chosen because of their distinct physicochemical characteristics, including the residual cementitious phases of RCP, the aluminosilicate-rich nature of WCP, and the amorphous silica structure of obsidian, which provide a basis for directly comparing their effects on particle packing, pore structure, and matrix development under the same FC production conditions. For this purpose, different substitution ratios and mixture parameters were considered to evaluate the effects of aggregate type and replacement level on FC performance. The specimens produced within the experimental program were systematically characterized for CS, durability performance, thermal behavior, and microstructural properties supported by SEM–EDS and XRD analyses.

## 2. Materials and Methods

### 2.1. Materials

**Figure 1:**
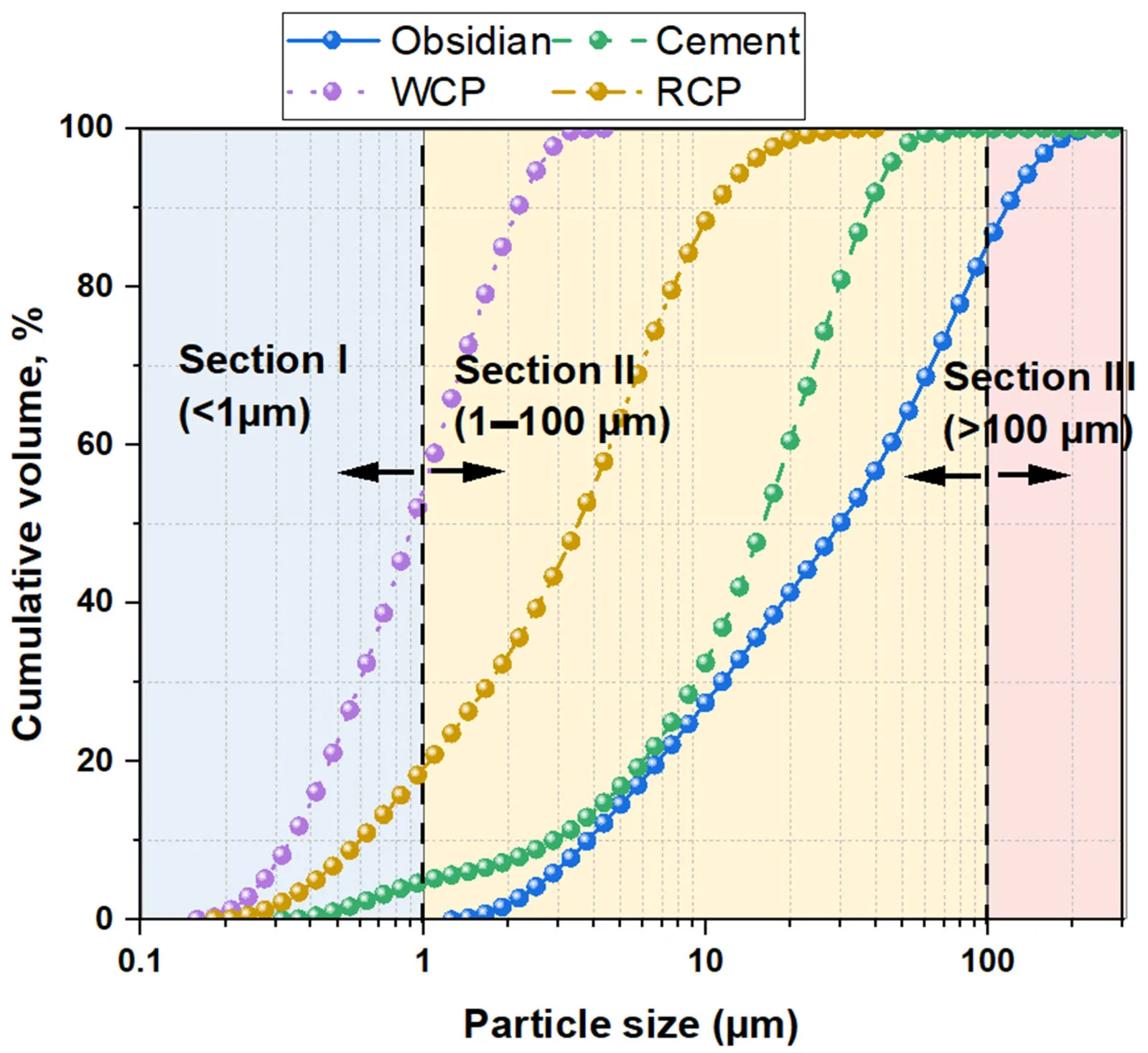
Cumulative volume distributions of obsidian, RCP, WCP, and cement.

CEM I 42.5 R class Portland cement was employed as the binder material in the production of FC. The cement conforms to the TS EN 197-1 [60] standard. Its chemical constitution was obtained by X-ray fluorescence (XRF) analysis, and the obtained data are given in Table 1. Cement particle gradation is shown in Figure 1.

In this study, RCP was obtained from Cevahir Ready-Mixed Concrete Plant. The RCP is composed of the standard concrete cube specimens with a CS of 20–30 MPa, which is the normal strength concrete used in the construction industry. The samples were mechanically cleaned of surface contaminants and then dried at ambient temperature. Then, the concrete specimens were recycled after cleaning and drying processes by crushing and grinding to obtain fine RCP particles. The RCP particle gradation is shown in Figure 1. The chemical constitution of the RCP used is shown in Table 1.

WCP used in this study was employed as an aggregate option alternative to natural sand in foam concrete mixtures. Ceramic-based wastes were obtained from production facilities as well as construction and demolition activities and were subjected to the same recycling and size-reduction process applied to concrete waste materials. The resulting WCP was then used to partially replace standard sand at specified proportions in the foam concrete mixtures. The WCP particle gradation is shown in Figure 1, and its chemical characteristics are shown in Table 1.

Obsidian was used as an alternative aggregate source, and the material was derived from the Çağırankaya region of the İkizdere region in Rize county. Obsidian particle gradation is illustrated in Figure 1. The chemical constitution of the obsidian used is shown in Table 1.

European Committee for Standardization (CEN) standard sand, adherent to the TS EN 196-1 [61] standard and supplied by Limak Cement, was used as the reference aggregate, consisting predominantly of silicon dioxide (approximately 98%).

The plant-based foaming agent was supplied by Kalzer Foam Concrete. Density of the material is 1.02 kg/L, so unit weight of concrete can be varied in the range of 200–1800 kg/m^3^.

The stability of the foam was preserved through the integration of HPMC. This reagent was obtained from Betakim Tekstil Sanayi ve Ticaret Limited Şirketi. HPMC was chosen to achieve stability in the foam in the wet concrete and also evenness in the size of the bubbles. The density of HPMC is 1.39 g/cm^3^.

### 2.2. Preparation of the Samples

FC mixtures were fabricated by the method of pre-formed foam. The bio-based foam agent was diluted to 2% with water and put into the foam tank. Then HPMC was added at 50% by weight of the foaming additive, and the mixture was homogenized. Foam was formed by a compressor operating at 3–6 bar to maintain the quality and stability of foam formation.

The standard sand was selected as the fine aggregate and substituted with RCP, WCP and obsidian powder at incorporation levels of 25%, 50%, and 100% on an equal-mass basis. This approach was preferred for its greater measurement precision compared to volume-based batching and to keep all other mix constituents strictly constant. Because the constituent materials have different densities, equal-mass replacement results in different aggregate volume fractions; therefore, the observed performance differences were interpreted by considering both the aggregate type and the resulting changes in the physical characteristics of the mixtures. First, the alternative aggregate materials were subjected to crushing using the jaw crusher, and then the crushing product was ground using the ball mill with a rate of 2 kg per hour. The cement paste was prepared by combining the dry materials and adding water and mixing for 2 min with an electric hand mixer with mixing attachment at 900 rpm. The mixture was mixed for another 2 min before the pre-formed foam was added. The mixture was mixed for another 2 min to ensure uniform distribution of foam in the cement mixture. Then the freshly mixed FC was cast into molds.

**Figure 2:**
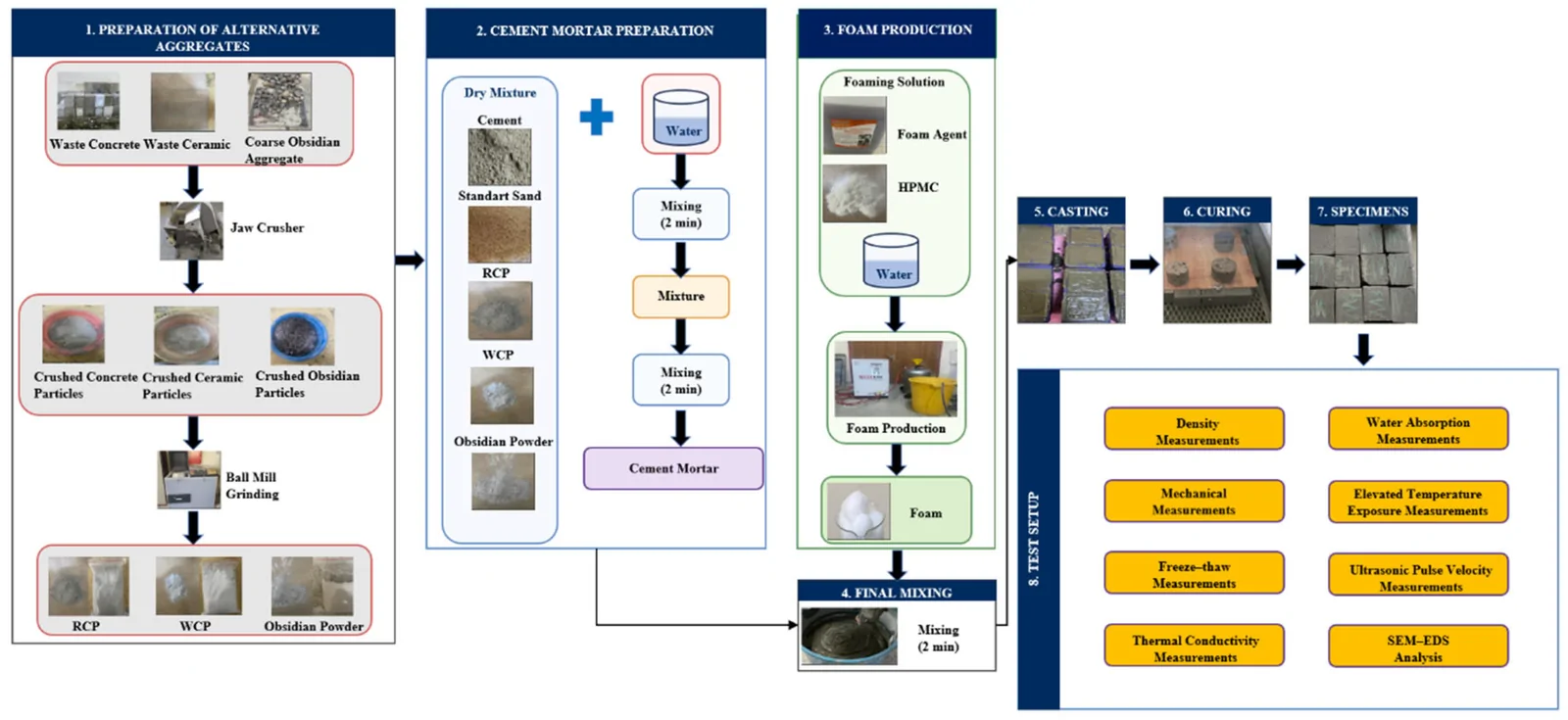
Conceptual diagram of the FC production process.

The samples were cured for three days in the laboratory environment, and after that, they were submerged completely in water at 23 ± 2 °C and 95% relative humidity until the designated testing ages. All specimens, regardless of the test type, were subjected to the same curing regime. Compressive strength was evaluated at 7 and 28 days, while the specimens for the other tests were tested at the corresponding designated age following the same curing procedure. The process diagram of the FC production procedure is shown in Figure 2.

For the mix design of this research, one control mix and three experimental series were used. The quantities of the binders, W/C ratios, and foams were kept the same in all cases in order to vary only the aggregates in the mix design. The mixture proportions are given in Table 2.

### 2.3. Methodology

The selected tests were intended to be a complementary assessment of the mechanical, physical, thermal and durability performance of FC. Mechanical performance and internal structural integrity were evaluated using CS and UPV, whereas physical characteristics and susceptibility to water penetration were assessed using density and WA. The TC was studied, as it is one of the main functional characteristics of FC. The mixtures were subjected to elevated temperature and freeze–thaw tests to evaluate their response to thermal exposure and repeated environmental cycles. For the elevated-temperature, freeze–thaw, SEM–EDS and XRD assessments, S1, S4, S7, and S10 were selected to provide a direct comparison between the reference mixture and the full-replacement mixture of each alternative aggregate type.

#### 2.3.1. Density Measurements

The density of the samples was computed following TS EN 12390-7 [62] using the mass and volume of 10 × 10 × 10 cm cube specimens. The specimen mass was determined using a high-precision balance, and the unit weight was subsequently calculated using Equation (1).

(1) $$\rho =\frac{m}{V}$$

where ρ is the density (kg/m^3^), *m* is the total mass of the prepared sample measured at the time of testing (kg), and *V* is the determined volume of the specimen (m^3^).

#### 2.3.2. WA Measurements

WA properties of the specimens were calculated following TS EN 12390-7 [62] after 28-day curing using cubic specimens with dimensions of 10 × 10 × 10 cm. Prior to evaluation, the samples were oven-conditioned at 105 ± 5 °C till an invariable mass was recorded, and their dry masses were documented. The specimens were then submerged in water for 24 h to achieve saturation, after which the saturated surface-dry masses were determined following the removal of free surface water. The WA values were computed using Equation (2).

(2) $$m=\left[\frac{M_{2}-M_{1\;}}{M_{1}}\right]\times 100\;$$

where *m* denotes the WA ratio (%), *M*_1_ represents the dry mass of the sample (kg), and *M*_2_ represents the saturated surface-dry mass (kg).

#### 2.3.3. Mechanical Measurements

**Figure 3:**
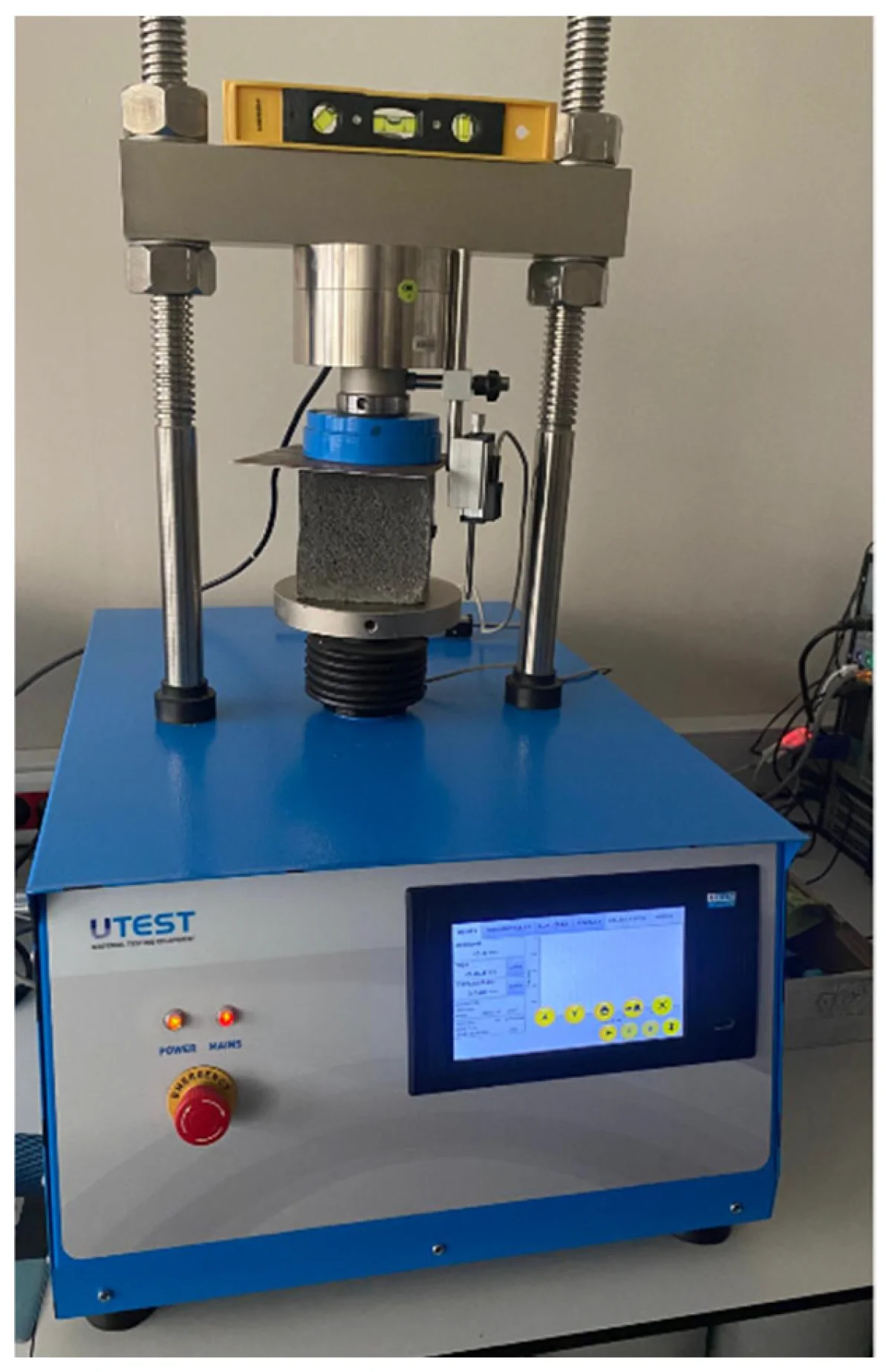
Experimental setup for the CS test.

CS tests were executed on the test specimens after 7 and 28 days of curing. For each series, 3 cubic samples with dimensions of 10 × 10 × 10 cm were assessed using a California Bearing Ratio (CBR) device. The tests were executed at a displacement rate of 5 mm/min, and a total of 60 prepared samples were crushed. The CS of each sample was computed as the proportion of the peak load attained during testing to the cross-sectional area, while the average value for each series was ascertained as the numerical average of three measurements. The CS of the test specimen was calculated using Equation (3) based on the failure load and sectional area. Figure 3 illustrates the CS test setup.

(3) $$f_{c}=\frac{F}{A_{c}}\left(\frac{N}{mm^{2}}\right)$$

where f_c_ is the CS of the test sample (N/mm^2^), F is the failure load (N), and A_c_ is the sectional area perpendicular to the loading direction (mm^2^).

#### 2.3.4. Elevated Temperature Exposure Measurements

**Figure 4:**
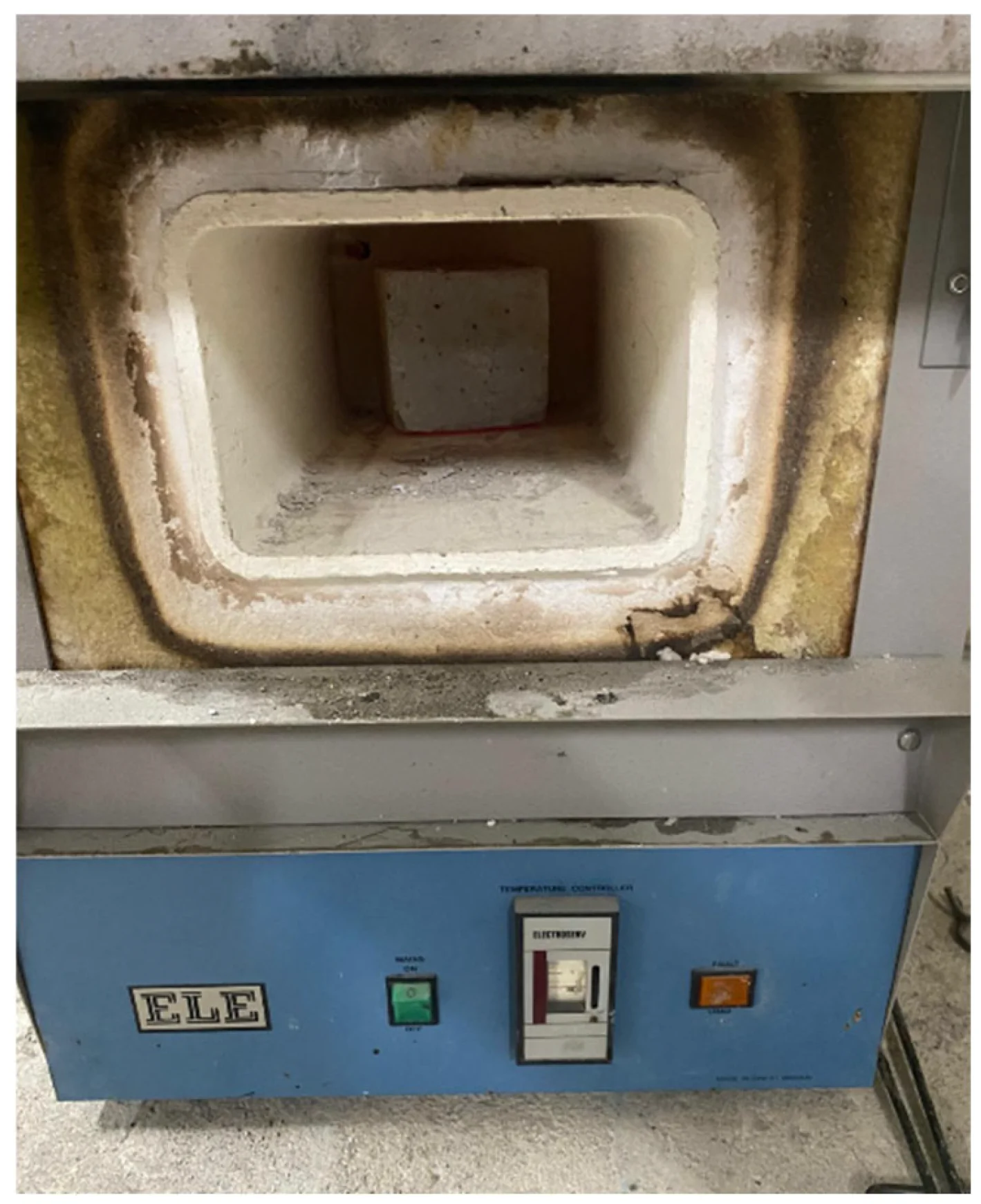
Programmable laboratory-scale electric furnace used for elevated-temperature exposure of the specimens.

An experimental investigation was undertaken to scrutinize the physical and mechanical effects of high temperature on the specimens. Cubic specimens with dimensions of 10 × 10 × 10 cm, aged at standard conditions for 28 days, were used in the study. The experiment was performed on S1, S4, S7 and S10 test specimens. After finishing the curing period, the test samples were conditioned at thermal conditions of 200, 400, 600, and 800 °C in a programmable electric furnace. The furnace reached the target temperature within 1 h, and the specimens were then kept at that temperature for 2 h. After thermal treatment, the test samples were left to cool naturally in the furnace to ambient conditions. The laboratory-scale furnace used for the elevated temperature treatment test is shown in Figure 4.

The samples were visually checked after cooling and surface cracking and color changes were recorded. Then, CS tests were performed to measure the strength losses due to high-temperature exposure.

#### 2.3.5. Freeze–Thaw Measurements

A controlled cycling test was performed to evaluate the freeze–thaw effect. The test was implemented on S1, S4, S7 and S10 test samples. Cubic specimens with dimensions of 10 × 10 × 10 cm, aged for 28 days at 23 ± 2 °C, were used in the test. Each cycle consisted of three successive stages. In the first stage, the test samples were gradually cooled from +20 °C to −20 °C over 4 h. In the second stage, the specimens were kept at −20 °C for 4 h to assure complete freezing. In the final stage, the specimens were gradually elevated from −20 °C back to +20 °C over 4 h to allow complete thawing. Accordingly, the total duration of one cycle was 12 h, and a sum of 16 cycles were applied throughout the experiment.

#### 2.3.6. UPV Measurements

UPV is a method employed to assess the internal soundness of cementitious materials in terms of voids, cracks, and structural discontinuities. In this work, assessments were undertaken on cubic specimens with dimensions of 10 × 10 × 10 cm aged for a period of 28 days at 23 ± 2 °C and 95% humidity level in accordance with ASTM C597-22 [63] using a Pundit PL-200 UPV tester. The pulse velocity values were determined using Equation (4).

(4) $$V=\frac{L}{T}$$

where V is the UPV (m/s), L is the center-to-center distance of the transmitter and receiver probes, or the specimen thickness (m), and T is the pulse transit time of the ultrasonic wave through the specimen (µs).

#### 2.3.7. TC Measurements

**Figure 5:**
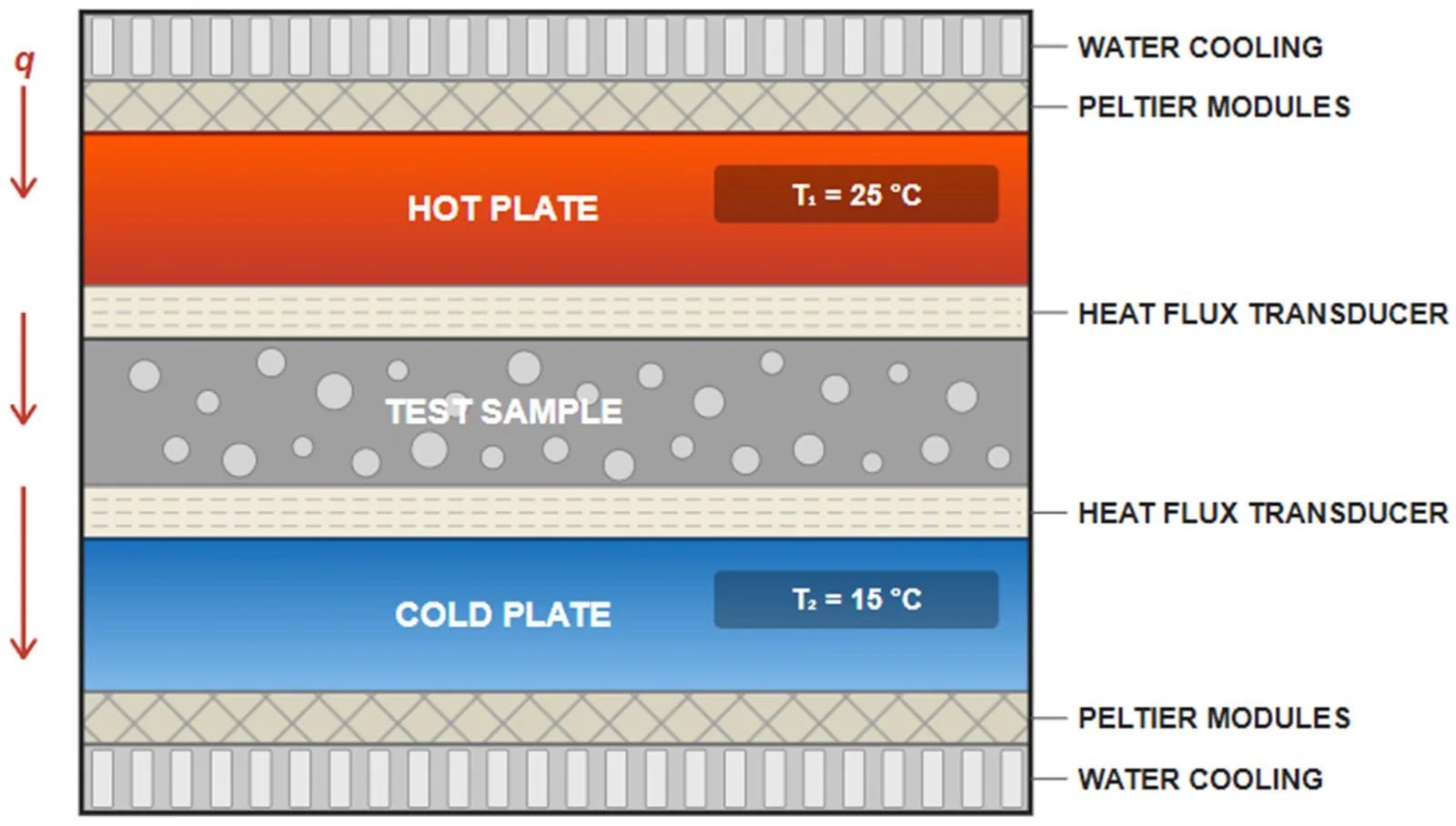
Graphical representation of the test configuration used for TC measurement.

TC of the specimens was observed by means of a steady-state heat flow method in compliance with ASTM C518-21 [64], founded on the principle of measuring the thermal flux transmitted through the test sample placed between two slabs maintained at various heat levels. The measurements were performed on prismatic specimens (5 × 30 × 30 cm) after 28 days of curing at 23 ± 2 °C using a LINSEIS HFM300 heat flow meter. The prepared test samples were located between the hot and cold slabs of the device and a constant temperature difference was kept across the thickness of the specimen during testing. The heat level gradient was held steady at 10 °C, the bottom plate at 15 °C and the top plate at 25 °C, as given in Figure 5. The TC of the specimens was computed in accordance with Equation (5).

(5) $$\lambda =\frac{\left(q\;\cdot \;d\right)}{\left(A\;\cdot \;∆T\right)}$$

where λ is the TC W/(m·K), q is the heat flux (W/m^2^), d is the test sample thickness (m), A is the specimen area (m^2^), and ∆T is the thermal gradient between the two plates (°C).

#### 2.3.8. XRF Analysis

The chemical constitution of cement, WCP, RCP and obsidian was assessed semi-quantitatively by XRF. Prior to analysis, the materials were finely milled to a grain size below 63 µm by means of a tungsten ring mill, followed by oven-conditioning at 105 ± 5 °C for 4 h. The L.O.I. was obtained by thermal treatment at approximately 1000 °C. After appropriate preparation, the samples were analyzed using a Rigaku ZSX Primus XRF spectrometer.

#### 2.3.9. SEM-EDS Analysis

SEM–EDS analysis was undertaken to study the internal architecture of the specimens, especially S1, S4, S7 and S10 types. The samples were taken from the FC and processed to form prism-shaped specimens with a thickness of about 1 cm. To improve the electrical conductivity of the specimens, their surface was gold coated. Microstructure assessment was executed employing a JEOL JSM-6610 electron microscope operated at an electron beam voltage of 15 kV.

#### 2.3.10. XRD Analysis

Powdered samples from the S1, S4, S7, and S10 mixtures were oven-dried and subsequently milled to a particle size of less than 40 μm with a ring mill. XRD analyses were performed with a Rigaku SmartLab X-ray diffractometer (Rigaku Corp., Tokyo, Japan) equipped with a D/teX Ultra 250 detector using Cu-Kα radiation in the 2θ range of 4–90° at the Recep Tayyip Erdoğan University Central Research Laboratory. The instrument was operated at 40 kV and 30 mA, with a scan speed of 3.03°/min and a step width of 0.02°.

## 3. Results

In the present study, samples of FC were manufactured by replacing the standard sand with obsidian, RCP, and WCP. First, reference samples were produced using Portland cement, water, standard sand and pre-formed foam. Mixture compositions were then produced by replacing the standard sand with obsidian, RCP, and WCP at 25%, 50% and 100% ratios. All the samples obtained were tested for their physical, durability, thermal and mechanical features. The outcomes showed that the substitution of alternative aggregate materials had a direct effect on the inner architecture and behavior of the FC. Especially the changes in matrix structure with increased substitution ratio, which was decisive for the determination of the density, WA behavior and thus the mechanical properties achieved.

### 3.1. Density

**Figure 6:**
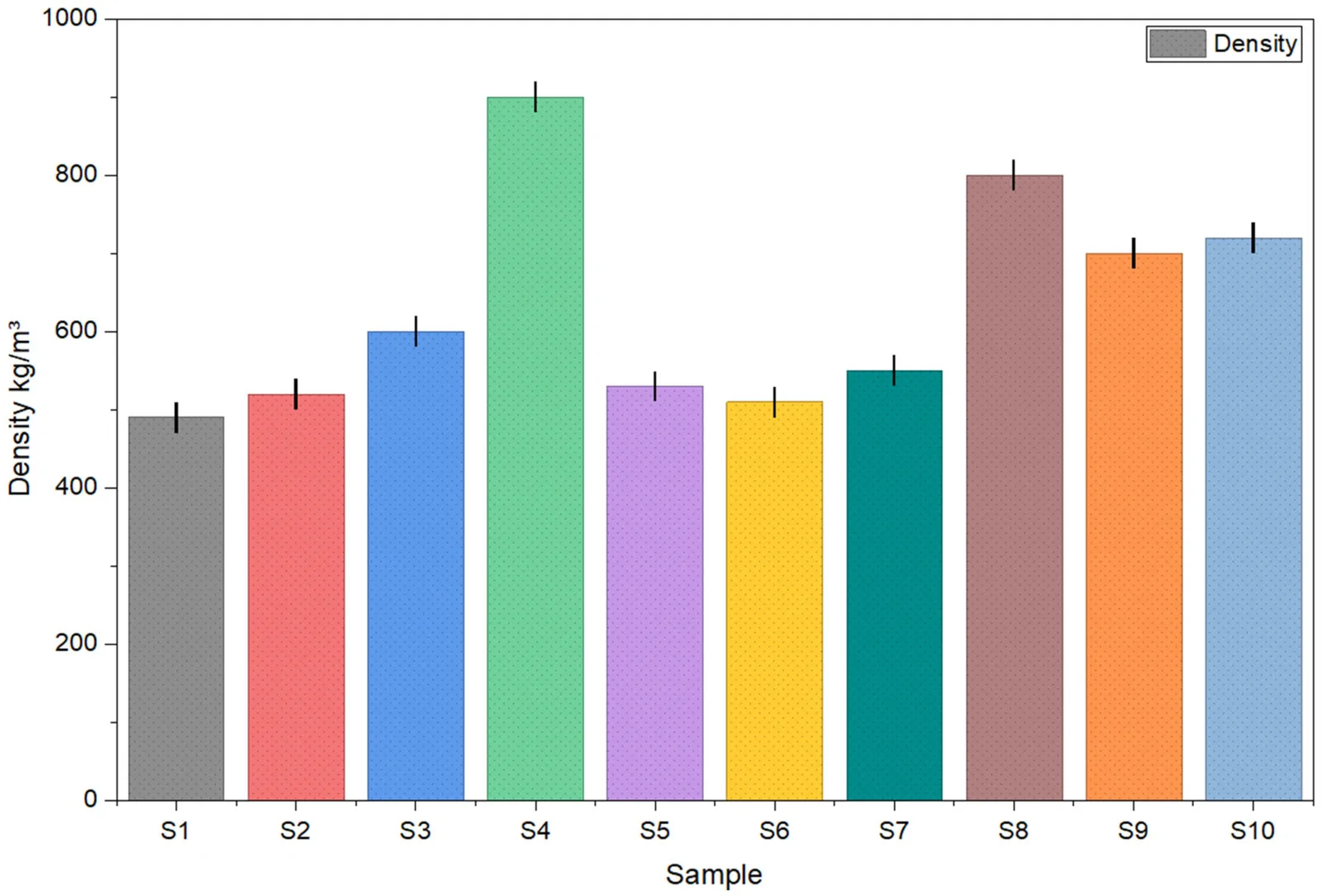
Variation in density with increasing alternative aggregate replacement ratio in FC mixtures.

Figure 6 presents the density tests performed on samples prepared by replacing the standard sand with obsidian powder, WCP and RCP. The reference sample (S1) of 100% standard sand was used as the unit weight 490 kg/m^3^. The density of the series of obsidian showed a gradual improvement with the increase in substitution ratio; the densities of specimens S2, S3, and S4 were 520, 600, and 900 kg/m^3^, respectively, which showed an increase of about 6.1%, 22.4%, and 83.7% compared to the reference sample. In the WCP series, the density values were 530 kg/m^3^ (S5), 510 kg/m^3^ (S6) and 550 kg/m^3^ (S7) which represented increases of about 8.2%, 4.1% and 12.2%, respectively. In the RCP series, the corresponding values were 800 kg/m^3^ (S8), 700 kg/m^3^ (S9) and 720 kg/m^3^ (S10) which meant increments of about 63.3%, 42.9% and 46.9%, respectively. In all the modified series, the density increased in comparison with the reference sample, and the highest value was obtained for the S4 specimen. This increase is due to the particle characteristics of the alternative aggregates. The smaller particles are responsible for better void filling in the inner-matrix, which results in a condensed internal structure.

### 3.2. Water Absorption

**Figure 7:**
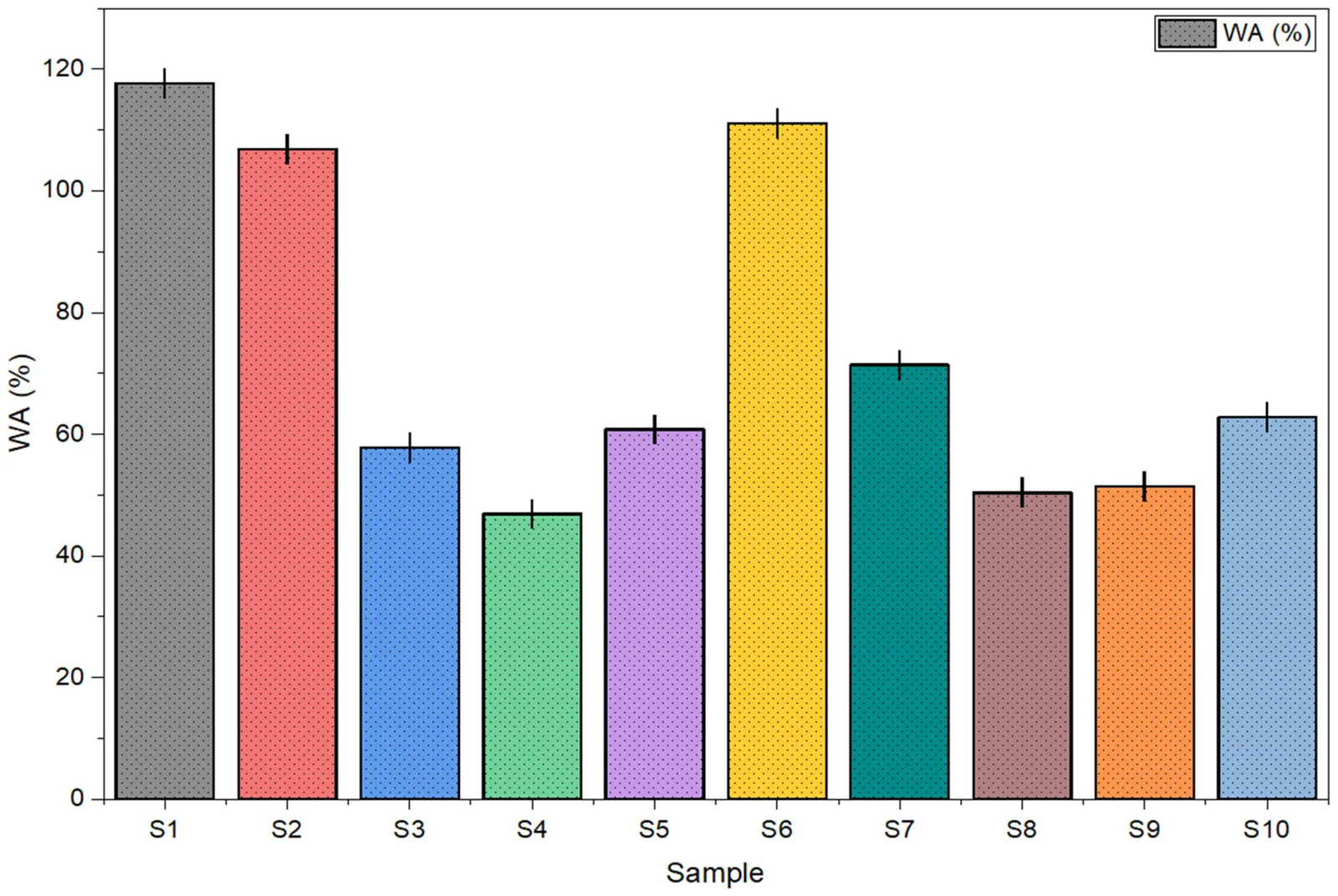
Comparative WA behavior of FC mixtures incorporating alternative aggregate.

The recorded WA results were compared with a reference sample (S1) with 100% standard sand. The relevant results are given in Figure 7. The WA rate of the reference sample was found to be 117.7%. In the mixtures with the powder of obsidian, a strong decrease in WA values with increasing substitution ratio was indicated. In this regard, the WA rates were obtained as 106.9%, 57.8% and 46.9% in the samples S2, S3 and S4, respectively. This decrease in WA was accompanied by the progressive increase in density observed across the obsidian series, indicating a clear relationship between the physical characteristics of the mixtures and their water absorption behavior. In the mixtures containing WCP, a variable WA behavior was observed with values of 60.8%, 111.1% and 71.4% for S5, S6 and S7, respectively, indicating a fluctuating response depending on the substitution ratio. In contrast, the mixtures containing RCP exhibited generally lower WA values compared to the reference sample, with recorded values of 50.4%, 51.4% and 62.8% for S8, S9 and S10, respectively, which can be associated with the residual cementitious phases and micro-filler effect of RCP particles.

### 3.3. Compressive Strength

**Figure 8:**
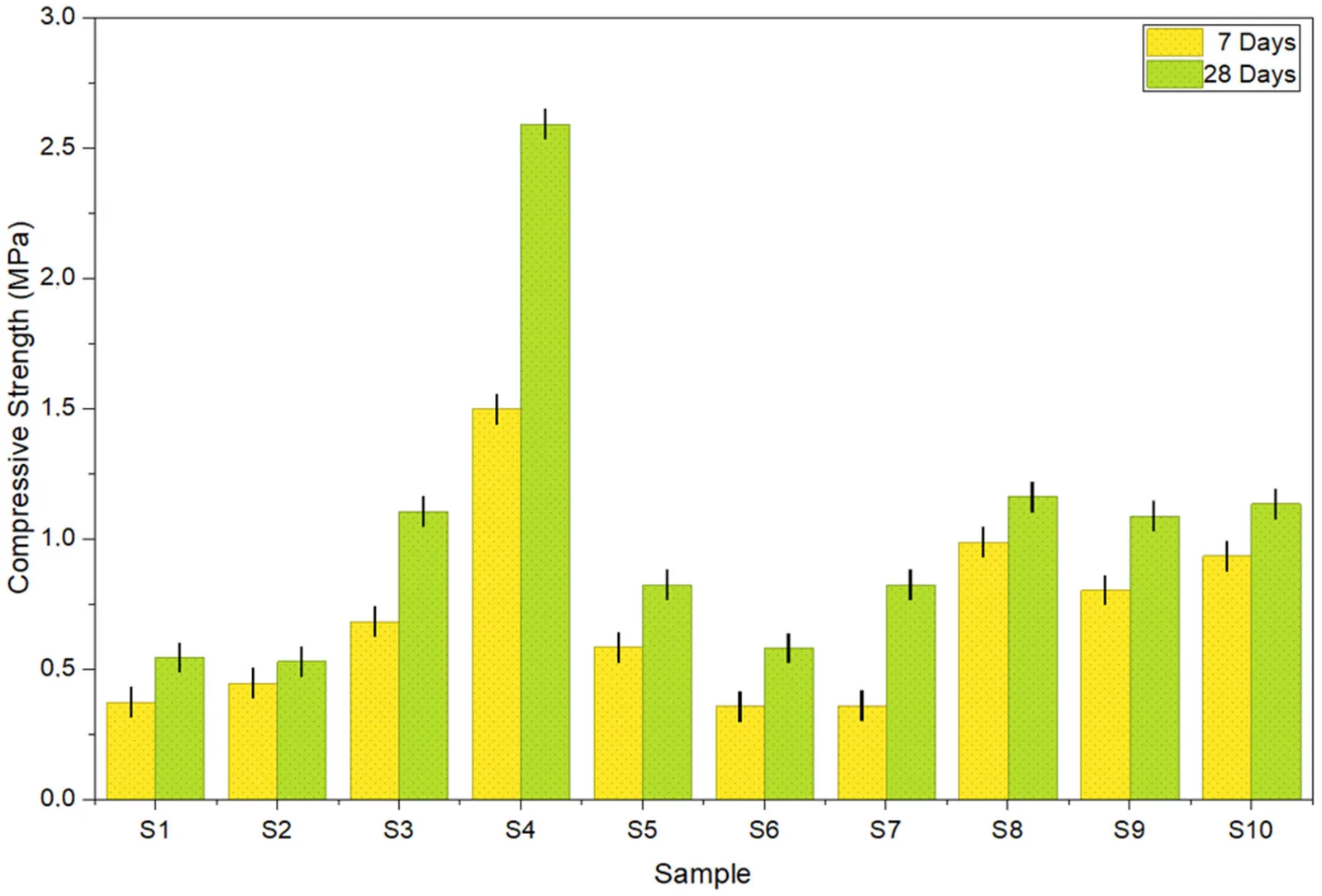
CS of S1–S10 samples at 7 and 28 days of curing.

The CS results presented in Figure 8 were compared with the S1 sample containing 100% standard sand, which was taken as a reference. For the S1 sample, the CS was 0.374 MPa at 7 days and 0.545 MPa at 28 days. The use of alternative aggregate materials resulted in a general alteration in the strength development of the mixtures. In this context, the S2 sample with 25% obsidian substitution reached 0.446 MPa at 7 days (+19.3%) while its 28-day strength was 0.530 MPa (−2.8%). The S3 sample with 50% obsidian substitution achieved 0.682 MPa at 7 days (+82.4%) and 1.104 MPa at 28 days (+102.4%). The S4 sample with 100% obsidian substitution reached 1.497 MPa at 7 days (+300.6%) and 2.590 MPa at 28 days (+375.2%). The increase in CS is consistent with the concurrent increases in density and UPV and the large decrease in WA with increasing obsidian replacement. The combined trends suggest an increase in matrix compactness and internal continuity, especially at higher replacement levels.

In the mixtures containing WCP, the S5 sample reached 0.584 MPa at 7 days (+56.2%) and 0.824 MPa at 28 days (+51.0%), the S6 sample recorded 0.357 MPa at 7 days (−4.6%) and 0.581 MPa at 28 days (+6.6%), and the S7 sample exhibited 0.361 MPa at 7 days (−3.5%) and 0.823 MPa at 28 days (+51.0%). In contrast, the RCP mixtures showed 0.986 MPa at 7 days (+163.6%) and 1.162 MPa at 28 days (+113.1%) for S8, 0.804 MPa at 7 days (+115.0%) and 1.087 MPa at 28 days (+99.4%) for S9, and 0.934 MPa at 7 days (+149.7%) and 1.134 MPa at 28 days (+108.0%) for S10. The observed variations are associated with the modification of the internal matrix structure, where changes in particle packing density and void distribution govern bulk density and consequently influence CS development.

### 3.4. Ultrasonic Pulse Velocity

**Figure 9:**
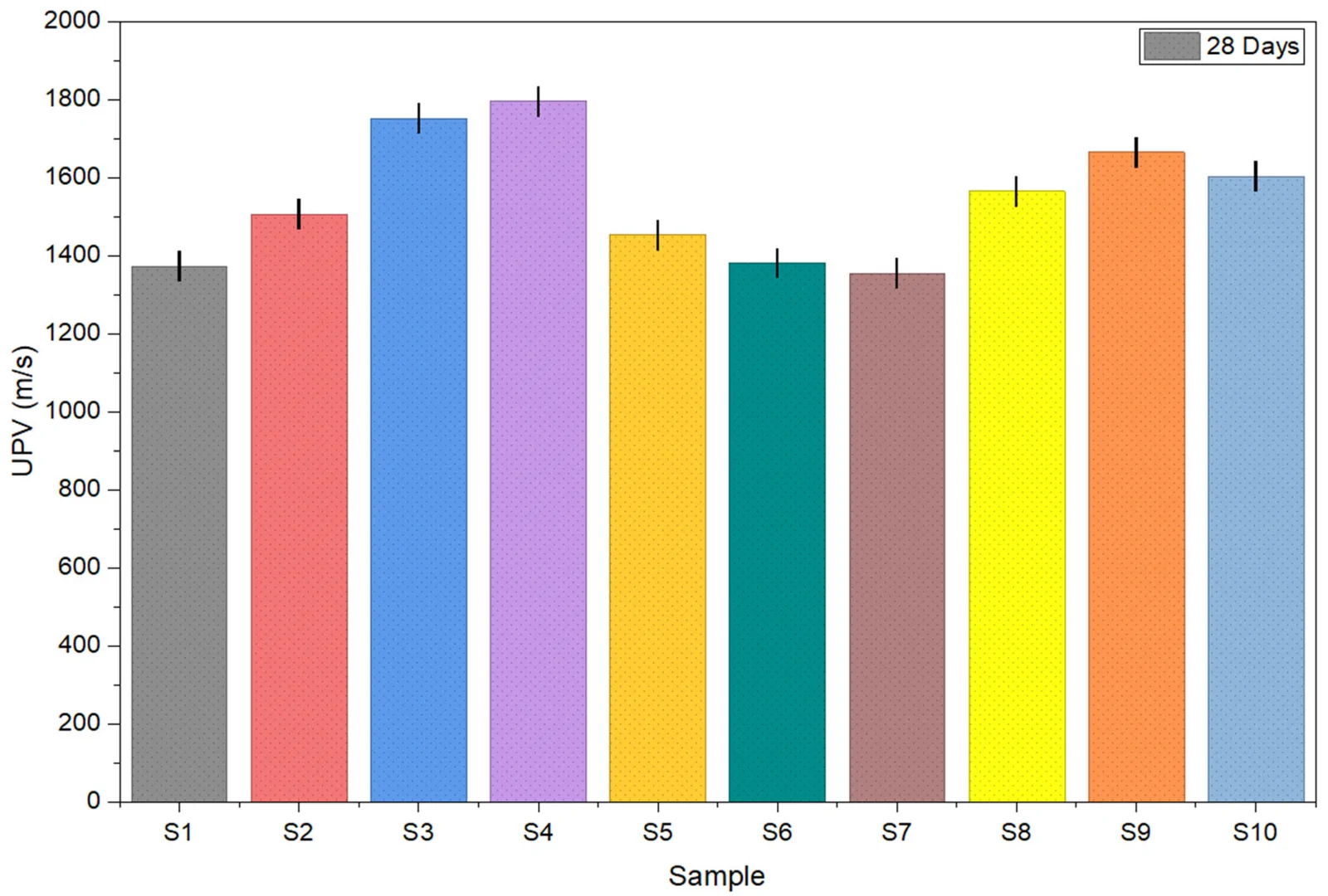
UPV of S1–S10 samples at 28 days.

The UPV results of FC specimens produced by partial and full replacement of the standard sand with obsidian powder, WCP and RCP are shown in Figure 9. The control specimen (S1) containing 100% standard sand was used as the reference mixture and had a UPV value of 1372 m/s. The UPV value in the mixtures with obsidian powder increased to 1506 m/s in S2 (+9.8%), to 1751 m/s in S3 (+27.6%) and as high as 1795 m/s in S4 (+30.8%), which is the best performance among all specimens. For the mixtures with WCP aggregates, the UPV value was obtained as 1453 m/s in S5 (+5.9%) and S6 reached 1381 m/s (+0.7%). In S7, with 100% WCP aggregate, UPV value decreased to 1355 m/s (−1.2%), lower than that of control specimen. For mixtures with RCP aggregates, the S8 recorded the UPV value of 1565 m/s (+14.1%), S9 1664 m/s (+21.3%) and S10 1603 m/s (+16.8%), all above the control mixture. In general, the highest UPV value was recorded for S4 at 28 days of curing while the lowest value was recorded for S7. The results show that the influence of alternative aggregates on ultrasonic wave propagation depended on the aggregate type and replacement level, with the most pronounced improvement observed in the obsidian series. The increase in UPV values in the obsidian mixtures is consistent with the concurrent increases in density and CS and the decrease in WA.

### 3.5. Thermal Conductivity

**Figure 10:**
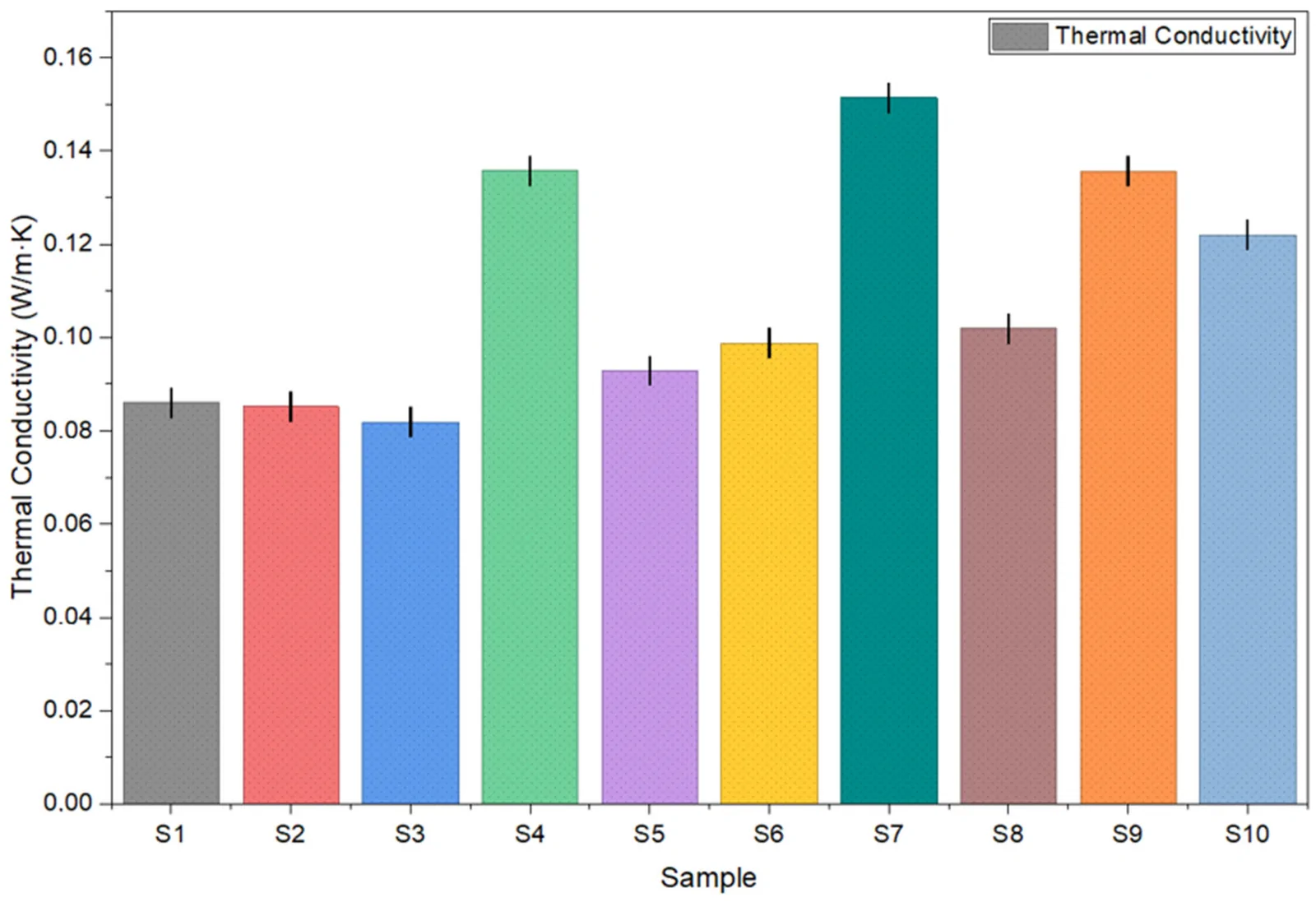
TC of FC specimens (S1–S10).

TC is largely controlled by a complex interplay of pore structure, density, aggregate properties, binder type and foaming agent and is a fundamental parameter in the assessment of thermal efficiency [13]. The measured TC values of the investigated specimens are presented in Figure 10. The S1 specimen was selected as the reference mixture having a TC value of 0.08606 W/(m·K). In the samples with obsidian powder, the reduction was noted at low replacement level, where TC values for S2 and S3 were 0.08520 W/(m·K) (−0.99%) and 0.08185 W/(m·K) (−4.9%), respectively, compared to S1. However, at full replacement a significant increase was observed, with S4 reaching a TC value of 0.13574 W/(m·K) (+57.7%) in comparison to the reference specimen. The TC values for the mixtures with WCP were 0.09287 W/(m·K) for S5 (+7.9%), 0.09875 W/(m·K) for S6 (+14.8%) and 0.15139 W/(m·K) for S7 (+75.8%) indicating a significant increment at full substitution. The TC values of S8 were 0.10187 W/(m·K) (+18.5%) in the RCP mixtures, S9 were 0.13563 W/(m·K) (+57.6%), and S10 were 0.12201 W/(m·K) (+41.8%), all higher than the reference specimen. Overall, the results suggest that lower obsidian replacement ratios can slightly improve the thermal performance, whereas full obsidian replacement causes a significant increase in TC. In contrast, WCP showed a progressive increase in TC, while RCP exhibited a non-monotonic response with replacement level. Such behavior is representative of a significant change in the thermal response of the matrix at higher replacement levels. The variations observed in TC are in agreement with the combined effect of aggregate type, replacement level and the resultant changes in density and internal matrix characteristics. The results show that the thermal performance of FC is significantly influenced by the type and percentage of alternative aggregates and the behavior of obsidian was comparatively better at lower replacement levels.

### 3.6. Elevated-Temperature Performance

**Figure 11:**
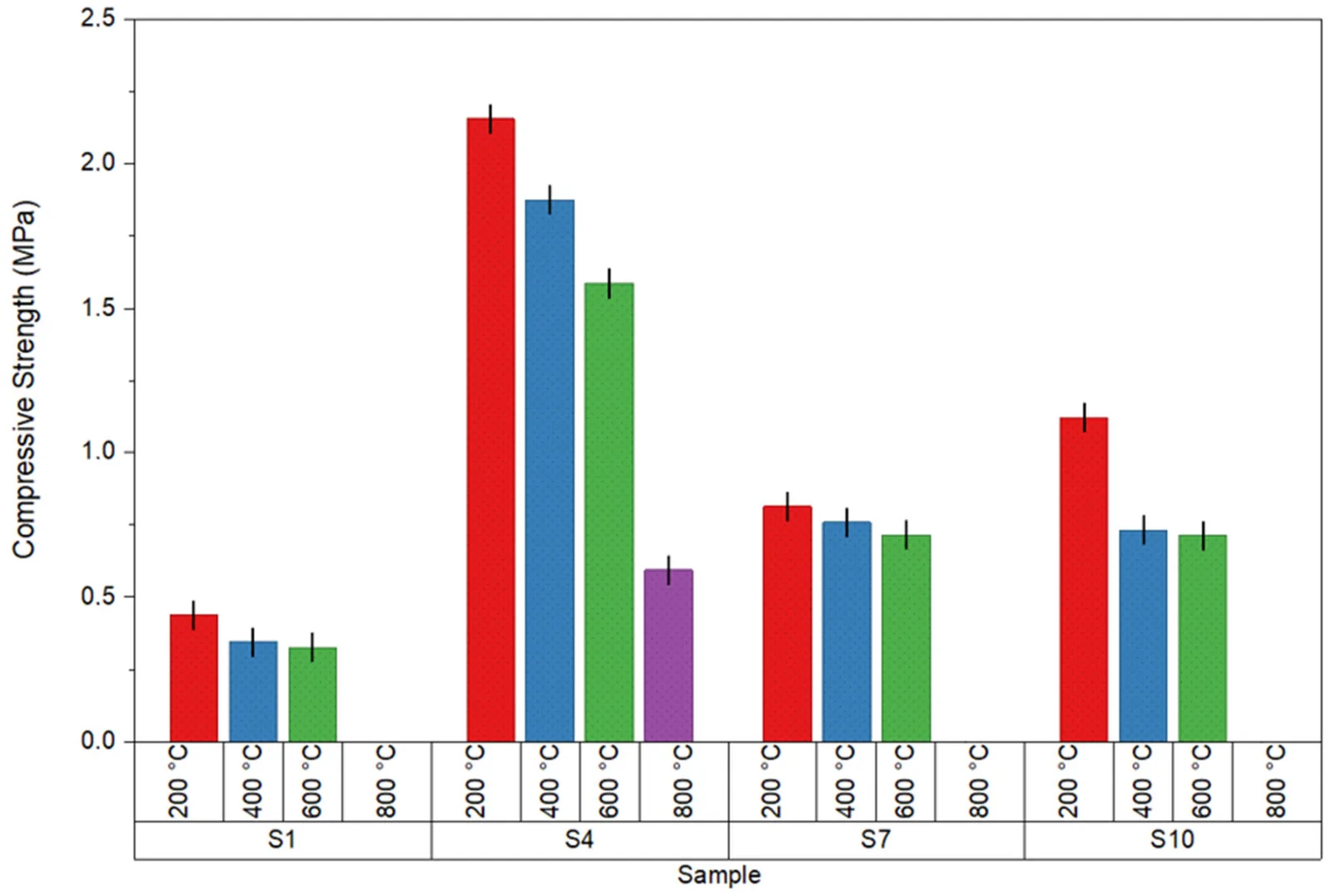
Residual CS of S1, S4, S7, and S10 samples at different temperatures.

The test specimens were thermally treated at 200 °C, 400 °C, 600 °C and 800 °C for 2 h and cooled down and tested for CS. The evaluation was conducted on S1, S4, S7 and S10 samples. Figure 11 shows the CS variation with temperature. The CS of the reference sample S1 decreased slowly with increasing temperature. The CS was 0.4368 MPa at 200 °C and was reduced to 0.3438 MPa at 400 °C and to 0.3250 MPa at 600 °C. This reduction is attributed to degradation of hydration products, evaporation of physically bound water and microcrack formation due to thermal exposure. Above 400 °C, the calcium silicate hydrate (C–S–H) gel structure was thought to be more strongly degraded, leading to a progressive weakening of the matrix integrity. However, the load-bearing capacity of the reference sample was lost completely at 800 °C and no measurable CS was observed, while, sample S4 (100% obsidian powder) exhibited the highest CS values at all temperature levels. The CS decreased from 2.1569 MPa at 200 °C to 1.8753 MPa at 400 °C and 1.5868 MPa at 600 °C then decreased to 0.5936 MPa at 800 °C. The results indicate that thermal degradation occurs at all stages, although the obsidian-based matrix has a relatively higher structural stability. The enhanced performance is associated with the amorphous and silica-rich property of obsidian, resulting in a denser and more thermally resistant microstructure.

However, the continuous growth of microcracks under high temperature is associated with continuous strength degradation. Sample S7 with 100% WCP also showed a gradual decrease in CS with temperature. The CS values decreased from 0.8142 MPa at 200 °C to 0.7593 MPa at 400 °C and 0.7149 MPa at 600 °C. The total loss of strength was observed at 800 °C. However, such a decrease was relatively small, suggesting that the WCP can provide better thermal stability and damage resistance against temperatures up to 600 °C. The CS of sample S10 with 100% RCP decreased from 1.1226 MPa at 200 °C to 0.7322 MPa at 400 °C and 0.7137 MPa at 600 °C, and lost all the strength at 800 °C. The significant decrease, especially in the range of 200 °C to 400 °C, suggests that the presence of attached old mortar and the deteriorated interfacial transition zone contributes to the development of thermal deterioration.

In summary, the results indicate that all mixtures exhibit a loss of strength with temperature but the rate and extent of degradation are dependent on the type of aggregate. The mixture with obsidian (S4) showed the highest thermal stability followed by WCP (S7). RCP (S10) showed relatively higher sensitivity towards increased temperatures. These results indicate that the high-temperature response is influenced by the characteristics of the alternative aggregate and the resulting matrix structure.

**Figure 12:**
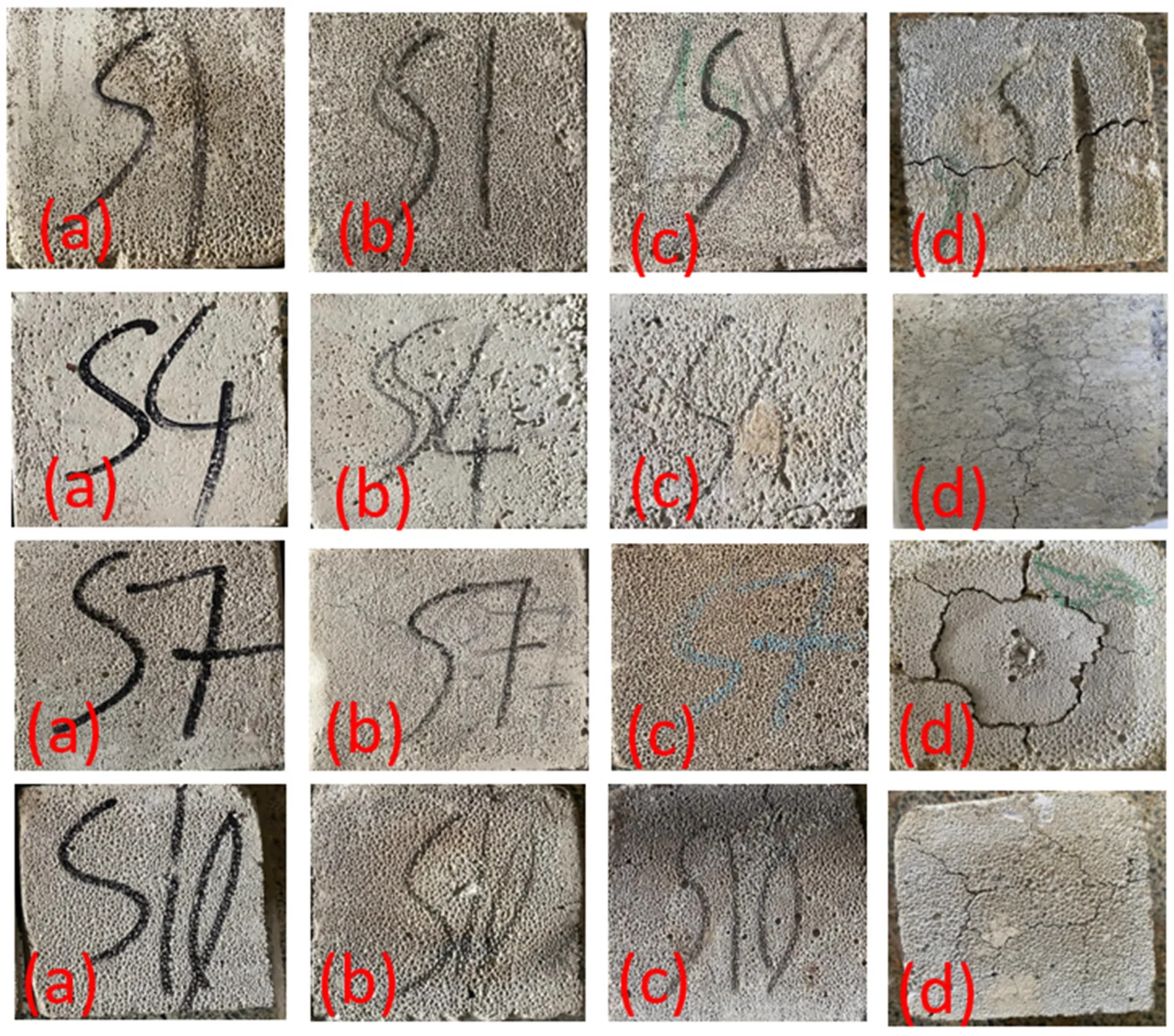
Evolution of surface morphology of S1, S4, S7, and S10 samples after exposure to elevated temperature levels: (**a**) 200 °C, (**b**) 400 °C, (**c**) 600 °C, (**d**) 800 °C.

The damage development in samples S1, S4, S7 and S10 with the increase in temperature exposure is shown in Figure 12. All specimens showed a thermally induced damage mechanism, characterized by progressive microcrack nucleation, propagation and disintegration of the matrix, with a transition from surface-dominated damage at lower temperatures to bulk structural damage at higher temperatures. In S1, microcracks were initiated as localized surface defects at 200 °C and 400 °C, while crack coalescence was observed at 600 °C due to dehydration of hydration products and progressive breakdown of the C-S-H gel network, leading to complete structural collapse at 800 °C. In contrast, S4 showed better thermal stability, where slight microcrack formation at 200–400 °C evolved to moderate cracking at 600 °C and even retained residual structural continuity at 800 °C, which is due to the thermally stable amorphous silica-rich framework of obsidian that improves the packing density of the matrix and postpones crack percolation.

S7 exhibited stable behavior up to 600 °C with the appearance of surface microcracks and localized color alteration and then showed accelerated crack propagation and surface degradation at 800 °C as the thermal stability limits were exceeded. Similarly, S10 exhibited a significant sensitivity to intermediate temperatures, where the rapid formation of cracks after 400 °C is associated with the degradation of adhered old mortar and progressive weakening of the interfacial transition zone, leading to unstable crack growth under thermal stress. Overall, the results indicate that the extent of surface cracking and structural deterioration increased with temperature and varied depending on the aggregate type and resulting matrix characteristics, as clearly illustrated in Figure 12.

### 3.7. Freeze–Thaw Performance

**Figure 13:**
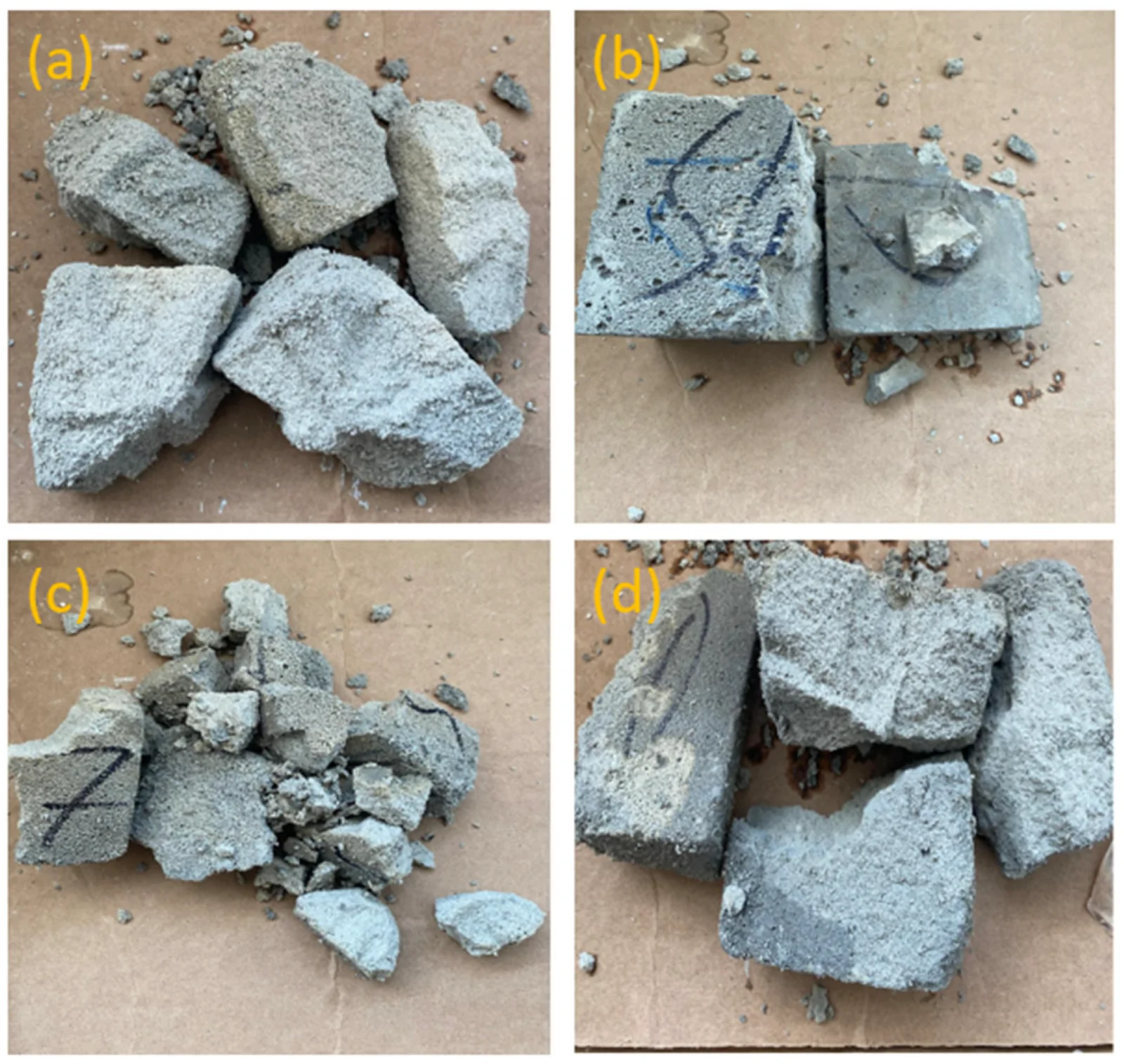
Surface deterioration of samples after freeze–thaw cycles: (**a**) S1, (**b**) S4, (**c**) S7, and (**d**) S10.

A total of 16 freeze–thaw cycles were applied to the specimens after 28 days of curing, and the physical deterioration that occurred upon completion of the cycles was evaluated. The assessment was performed on the reference specimen (S1) and the mixtures in which standard sand was fully replaced with obsidian powder (S4), WCP (S7), and RCP (S10). It should be noted that specimen S7 was removed from the freeze–thaw chamber after the 10th cycle due to severe deterioration and disintegration. The post-cycle appearances of the specimens subjected to repeated freeze–thaw exposure are presented in Figure 13. Distinct differences in physical performance were observed depending on the aggregate type used. Among all specimens, S4 containing obsidian powder largely preserved its structural integrity after 16 cycles and maintained a compact and coherent appearance. This observation is consistent with the superior CS performance of the same mixture and suggests that obsidian powder contributes to the development of a denser microstructure with lower pore connectivity at the aggregate–paste interface. This structure is expected to limit water ingress and internal damage due to ice formation in freezing conditions.

S1, S7 and S10 specimens showed a complete disintegration after exposure to freeze–thaw with severe deformation of the original specimen geometry. Moreover, the premature failure of S7, which lost its integrity after only 10 cycles, indicates the limited freeze–thaw durability of the WCP-containing mixture although it is relatively stable under high-temperature conditions. Similarly, the significant degradation observed in S10 suggests that the porous old mortar phase and weakening of the interfacial transition zones contribute to damage accumulation. In general, the results indicate that the aggregate type is an important factor controlling the freeze–thaw durability of FC. The better performance of S4 indicates that the integration of obsidian powder improves resistance to cyclic freezing and thawing under the applied test conditions by contributing to a denser and stronger internal structure.

### 3.8. SEM Analysis

**Figure 14:**
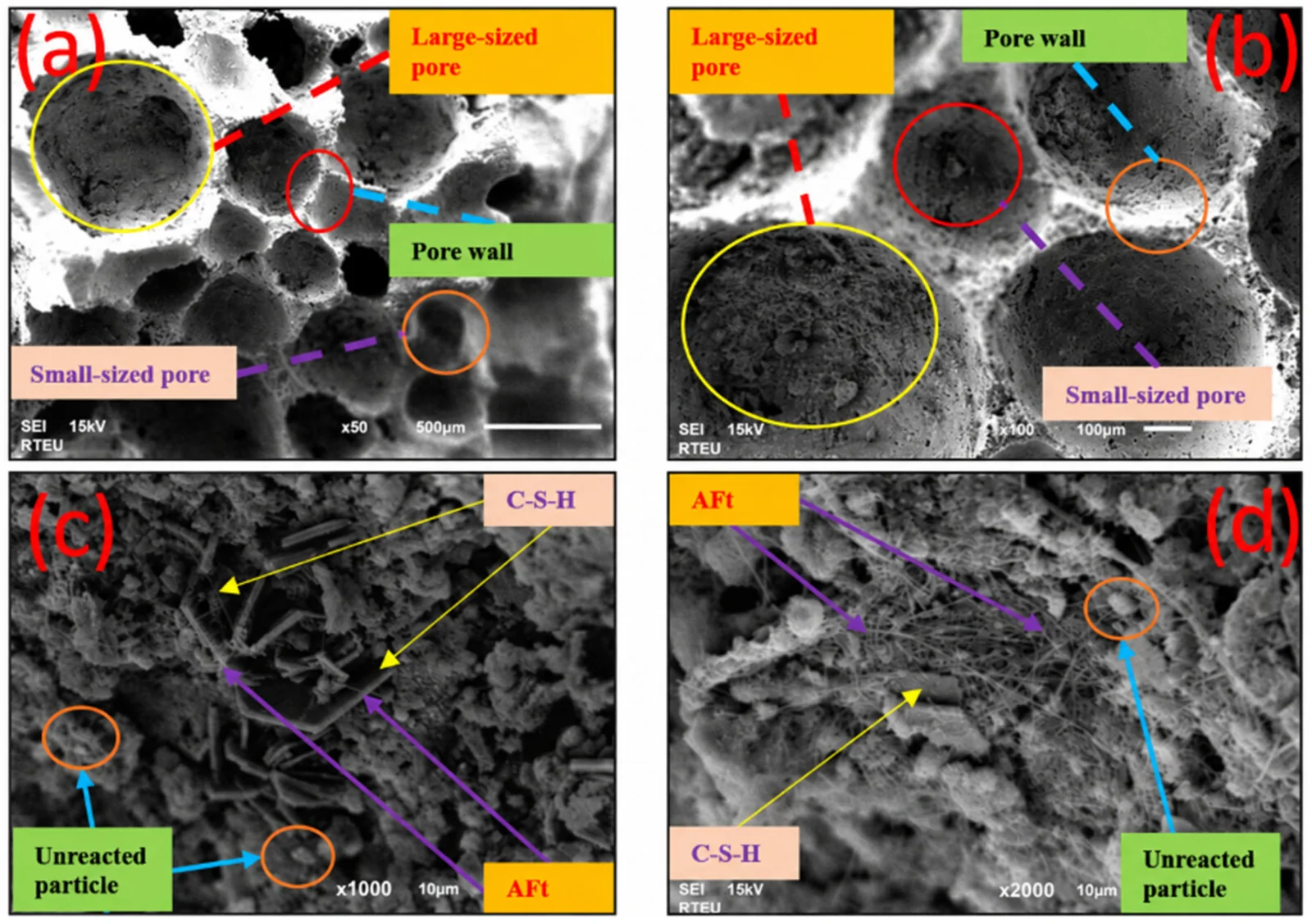
SEM images of sample S1: (**a**) 50×, (**b**) 100×, (**c**) 1000×, and (**d**) 2000×.

**Figure 15:**
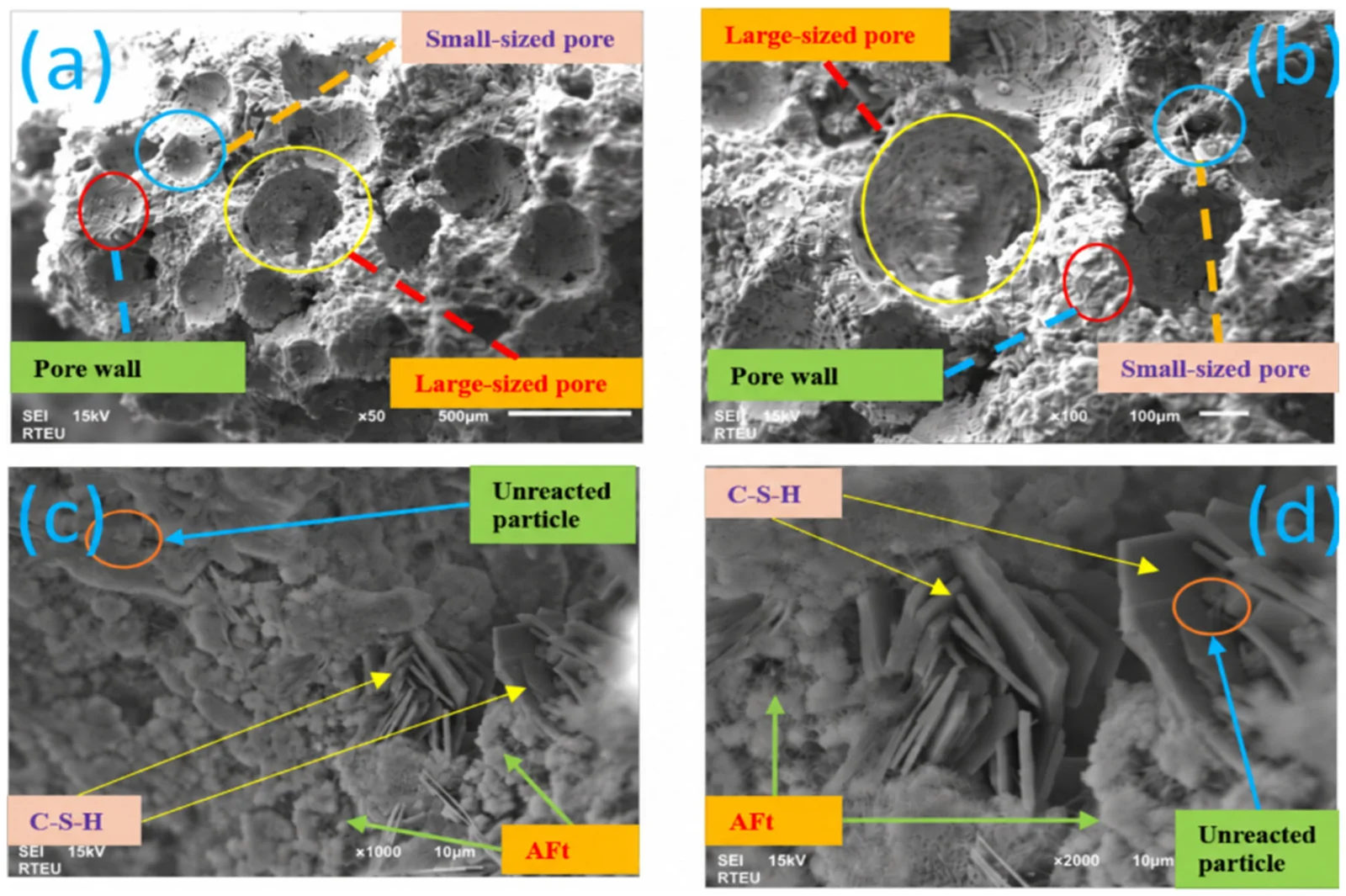
SEM images of sample S4: (**a**) 50×, (**b**) 100×, (**c**) 1000×, and (**d**) 2000×.

**Figure 16:**
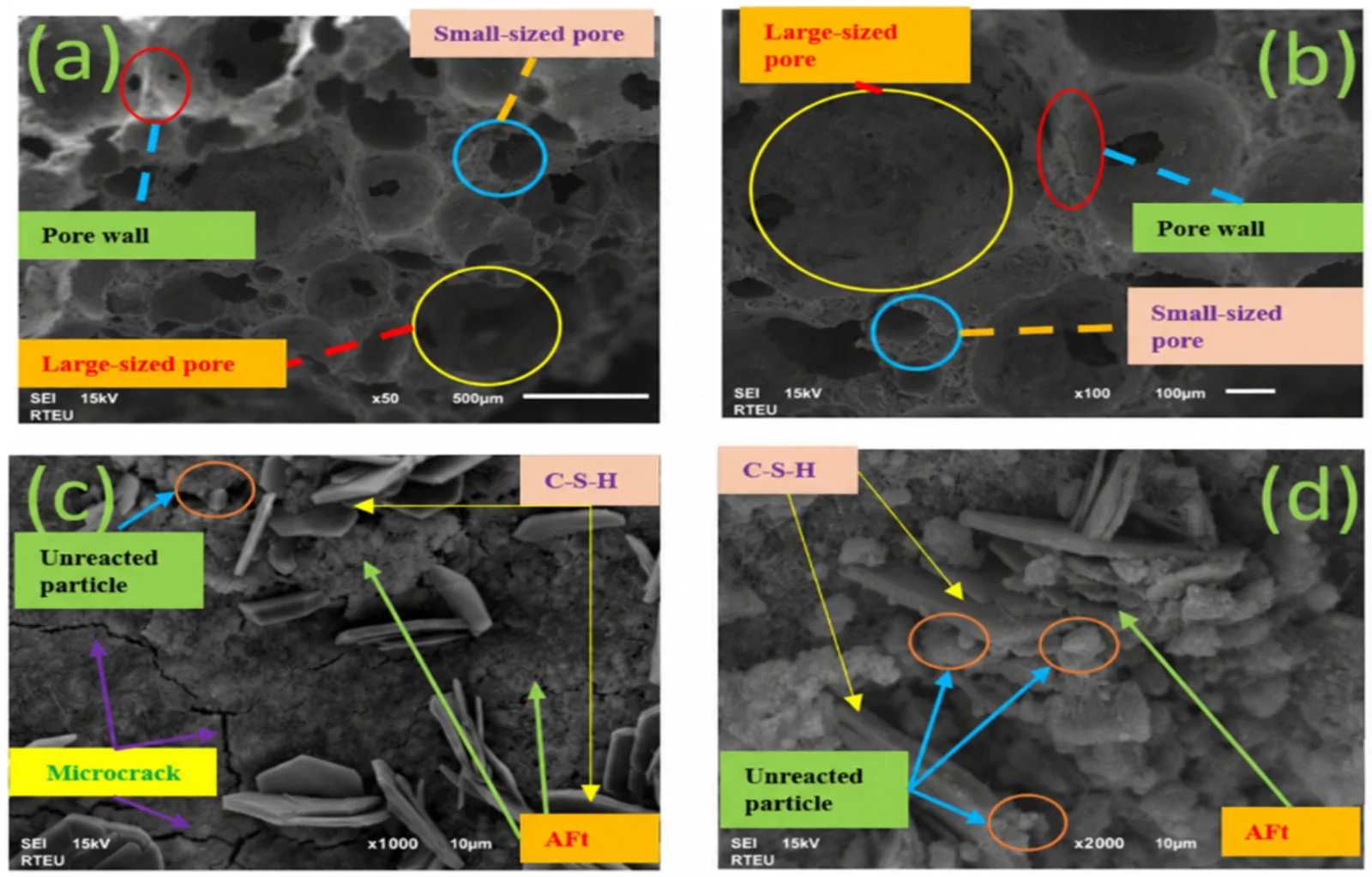
SEM images of sample S7: (**a**) 50×, (**b**) 100×, (**c**) 1000×, and (**d**) 2000×.

**Figure 17:**
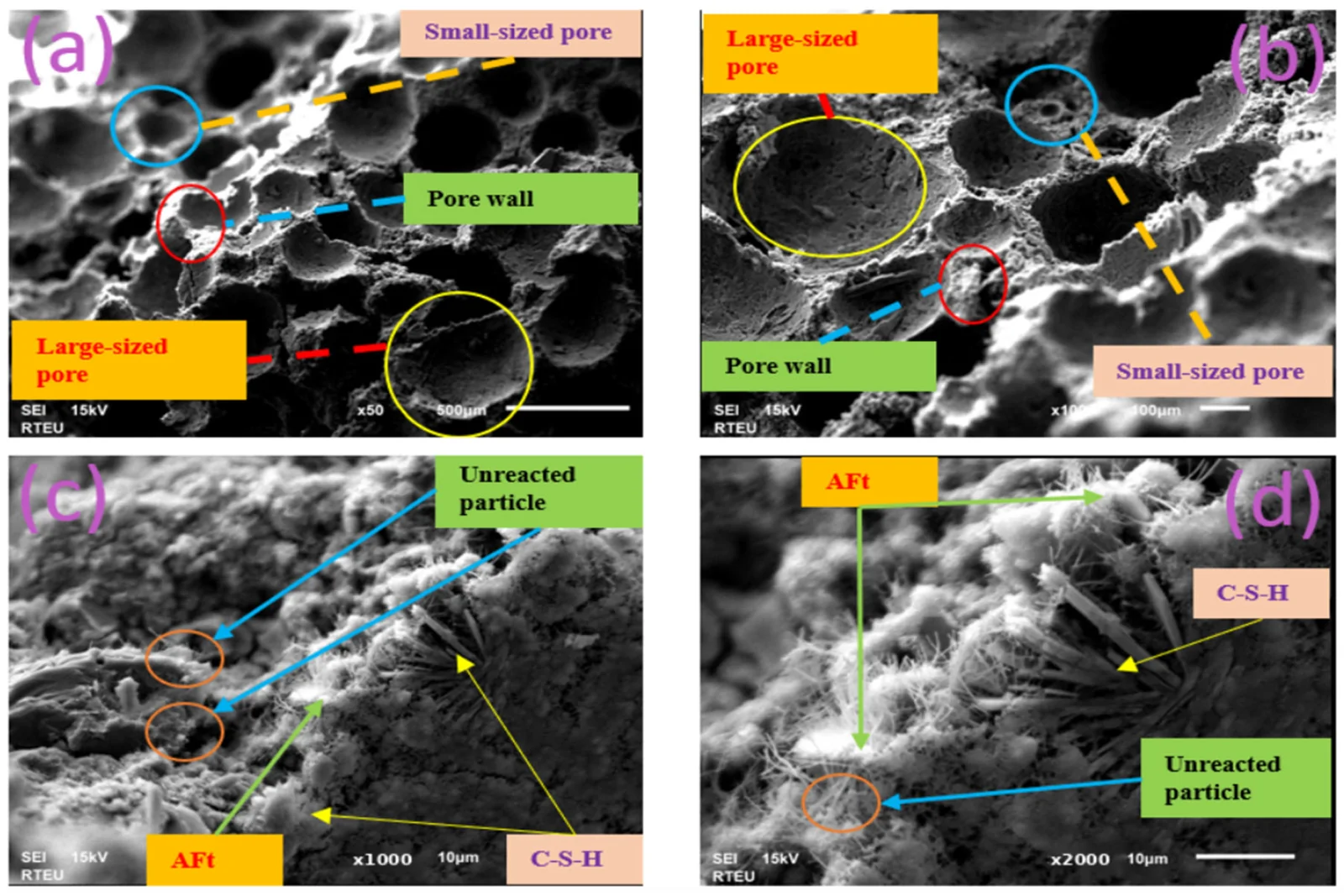
SEM images of sample S10: (**a**) 50×, (**b**) 100×, (**c**) 1000×, and (**d**) 2000×.

SEM analysis was performed to study the microstructural features including pore morphology, hydration products and matrix integrity at various scales in foam concrete specimens. SEM observations are very useful for the morphology and distribution of microstructural constituents and for a detailed assessment of the internal structure of the hardened matrix. SEM images of the S1, S4, S7 and S10 specimens are presented in Figure 14, Figure 15, Figure 16 and Figure 17, respectively. Each specimen was examined at four different magnification levels; 50× (a), 100× (b), 1000× (c) and 2000× (d). At low magnification levels (a and b), it is observed that all specimens exhibit the pronounced heterogeneous pore structure characteristic of foam concrete matrices. At 50× magnification, large and small pores with different sizes and pore walls can be generally distinguished. At 100× magnification, the pore size distribution becomes more evident, and the morphology and thickness of the pore walls can be examined in greater detail. It was determined that the pore structure shows a largely similar character in all specimens, and the heterogeneous distribution and variable pore sizes are consistently repeated from specimen to specimen.

At high magnification levels (c and d), important findings regarding the micro-scale internal structure of the matrix were obtained. At 1000× magnification, fibrous and gel-like structures belonging to the C-S-H phase and needle-like crystalline morphologies defined as the ettringite (AFt) phase were clearly detected in all specimens. In addition, unreacted particles were observed within the matrix; furthermore, the presence of microcracks was also noted in some regions. At 2000× magnification, these phases were visualized at higher resolution, revealing the distribution and interaction of the C-S-H gel structure, AFt crystals, unreacted particles and microcracks within the matrix in greater detail. In general, it is understood that similar microstructural features are observed in all S1, S4, S7 and S10 specimens. Despite differences in mix proportions, heterogeneous pore distribution, the presence of C-S-H and AFt phases, unreacted particles within the matrix and microcrack formations were observed consistently.

### 3.9. EDS Analysis

**Figure 18:**
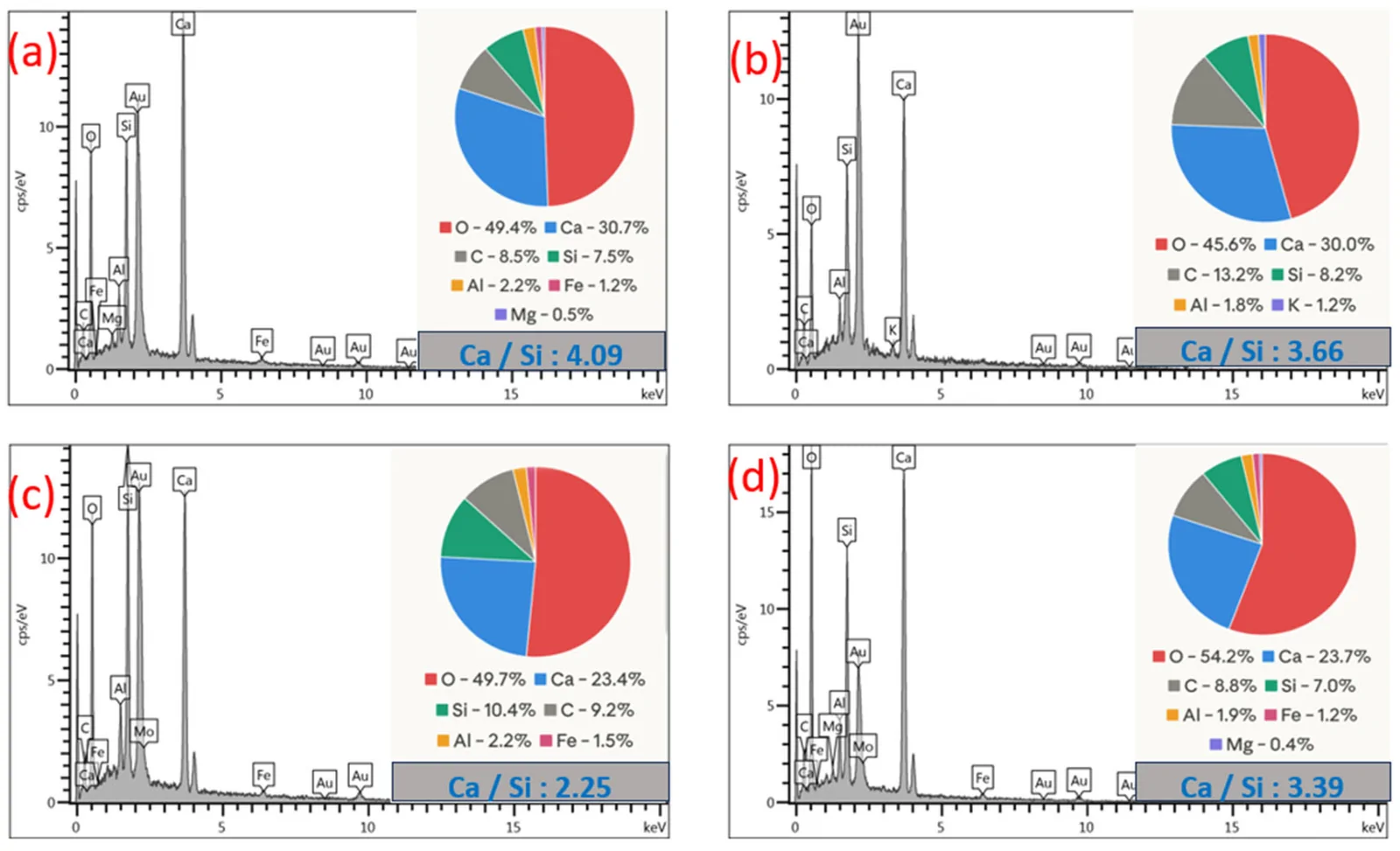
EDS spectra and elemental weight distributions of the investigated specimens: (**a**) S1, (**b**) S4, (**c**) S7, and (**d**) S10.

EDS analysis was performed to evaluate the elemental composition of the foam concrete specimens and to complement the SEM observations through chemical characterization of the matrix. The corresponding spectra and elemental compositions obtained from selected regions are presented in Figure 18. The elemental compositions obtained from the characteristic X-ray intensities collected from the surfaces of the specimens showed the presence of Ca, Si, O, C, Al, Fe and Mg in all the samples. The calculated Ca/Si ratios for S1, S4, S7 and S10 were found to be 4.09, 3.66, 2.25 and 3.39, respectively. The highest Ca/Si ratio was found for S1 and lowest Ca/Si ratio was found for S7. The dominance of calcium and the simultaneous presence of silicon and oxygen indicate the formation of C-S-H phases that are the main binding products in the hydrated matrix. The presence of Al, Fe and Mg shows the contribution of minor secondary phases and hydration products to the chemical composition of the specimens. The results of EDS support the SEM observations and give information about the microstructural features and distribution of the hydration products in the foam concrete matrix. Also, the differences in the Ca/Si ratios obtained in the samples suggest differences in the chemical composition of the hydration products, indicating the influence of the composition of the mixtures on the processes of hydration and pozzolanic reactions.

### 3.10. XRD Analysis

**Figure 19:**
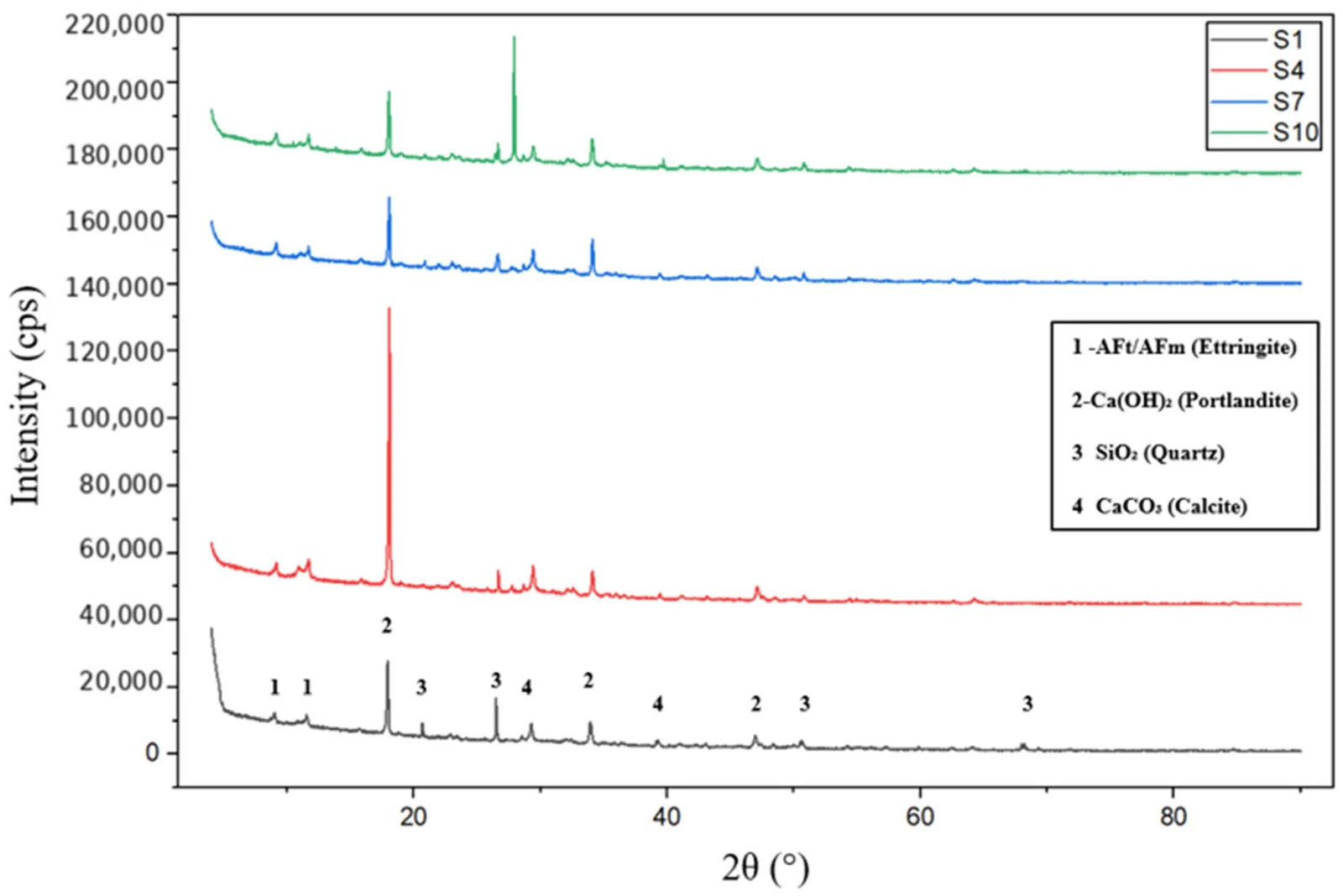
XRD patterns of S1, S4, S7, and S10 mixtures.

XRD patterns of S1, S4, S7, and S10 are presented in Figure 19. The main crystalline phases identified in the investigated mixtures were ettringite (AFt) and aluminate-ferrite-monosulfate (AFm) phases, portlandite (Ca(OH)_2_), quartz (SiO_2_), and calcite (CaCO_3_). All mixtures exhibited generally similar crystalline phase assemblages, although differences were observed in the relative intensities of the diffraction peaks. In particular, the portlandite reflection was pronounced in S4, while quartz- and calcite-related reflections were observed in the investigated mixtures. The diffraction patterns showed identifiable reflections mainly within the 2θ range of approximately 10–50°. A pronounced portlandite reflection was observed at approximately 18° 2θ, while the characteristic quartz reflection was observed around 26–27° 2θ. Calcite-related reflections were also detected, including the prominent region around 29–30° 2θ. Additional lower-intensity reflections associated with AFt/AFm and the identified crystalline phases were distributed throughout the investigated 2θ range. These variations indicate differences in the relative crystalline phase characteristics of the FC matrices containing the alternative aggregates.

## 4. Discussion

The overall results indicated that the behavior of FC containing obsidian powder, WCP and RCP could not be described by a single controlling factor but rather by the combined influence of aggregate characteristics, particle packing, and internal pore architecture, which was corroborated by the trends noticed in unit volume weight, CS, UPV, TC, WA, durability performance and microstructural observations. The unit volume weight increased in all modified mixtures compared to the reference sample with the most pronounced increase in the obsidian series, which was accompanied by a substantial reduction in WA. The density response of WCP was found to be fluctuating with respect to replacement ratio whereas RCP had relatively high density values due to the residual effect of mortar and filler. Overall, these results demonstrate that aggregate type and replacement level considerably influenced the physical characteristics of the mixtures. The CS results also confirm these findings by showing a significant increase in strength in the obsidian-based mixtures, particularly in S4, which suggests that the mechanical performance was not only due to aggregate replacement but also due to aggregate–matrix interactions. The RCP mixtures also showed important strength improvements and the WCP showed a more variable performance depending on the substitution rate. Simultaneously, the UPV values in the obsidian mixtures increased consistently with the replacement level, while those of the RCP mixtures remained above the reference level but did not increase monotonically, indicating differences in internal wave transmission behavior, while WCP showed limited trends at higher replacement levels. The TC results showed a replacement-dependent behavior, where lower obsidian replacement levels created a slight reduction in the TC, while higher replacement levels were generally associated with higher TC values, indicating that the thermal response was strongly dependent on aggregate type and replacement level.

The results were further confirmed with performance of durability at high temperature and freeze–thaw conditions. The obsidian-based mixture (S4) had better structural stability and retained residual integrity and strength after exposure to thermal and cyclic freezing–thawing, while the WCP and RCP mixtures showed earlier deterioration under the investigated conditions. These macroscopic trends were considered together with the SEM observations, which revealed heterogeneous pore structures, hydration products, unreacted particles, and local microcracks in all examined specimens. The XRD patterns additionally revealed generally similar crystalline phase assemblages consisting mainly of AFt/AFm, portlandite, quartz, and calcite, while differences were observed in their relative diffraction intensities. The comparatively higher density, CS, and UPV and lower WA of S4 indicate a more compact overall matrix response, while the SEM observations provide qualitative microstructural support for these macroscopic differences. Finally, the EDS results showed variations in local elemental composition, with Ca/Si ratios of 4.09 for S1, 3.66 for S4, 2.25 for S7 and 3.39 for S10. These variations indicate differences in the local chemistry of the hydration products associated with the different aggregate compositions. In this sense, the overall behavior of FC demonstrates that obsidian powder, WCP, and RCP influence the investigated properties through different physical and microstructural mechanisms, with obsidian providing the most consistent overall performance, RCP producing moderate improvements, and WCP exhibiting a more variable response depending on replacement ratio.

Beskopylny et al. [65] observed that incorporating fly ash together with micro- and nanosilica into FC lowered its density to 1142 kg/m^3^, while at the same time raising CS to 15.1 MPa, which corresponds to a 46.6% increase; this improvement was linked to intensified pozzolanic activity and the development of a more uniform pore system. In another study, Zhang et al. [66] observed a substantial increase in CS (29.73 MPa) in mixtures optimized by adding mullite powder, due to the generation of a dense microstructural network supported by C-S-H and AFt phases, demonstrating the significance of the matrix continuity in case of load transfer. Meanwhile, Yun-Lin et al. [67] reported that the CS decreased from 0.759 MPa to 0.227 MPa when the foam content rose from 59% to 87%, indicating the negative effect of higher porosity and larger pore sizes on the mechanical performance. Together, these studies suggest that pore structure characteristics and matrix integrity are the main factors controlling CS in FC, not just one compositional variable. Consistent with this general trend, the present study indicates that the addition of obsidian powder results in a continuous increase in the CS up to 2.590 MPa at 28 days in the S4 mixture, which is consistent with the simultaneous increase in density and UPV and the decrease in WA observed across the obsidian series. These combined results indicate improved matrix compactness and internal continuity with increasing obsidian replacement. Similarly, WCP-modified mixtures exhibit moderate strength gains at 28 days, particularly in S5 and S7 (0.824 MPa and 0.823 MPa), attributed mainly to particle packing and localized void redistribution rather than chemical reactivity, suggesting a predominantly physical mechanism. However, RCP mixtures showed a more considerable increase in CS with values of 1.162 MPa, 1.087 MPa and 1.134 MPa for S8, S9 and S10, respectively, which is consistent with their higher density and UPV values relative to the reference mixture, indicating improved internal continuity. Overall, the results obtained in this work are promising, as the alternative aggregate type and replacement level influence CS development. Among the alternative aggregates investigated, obsidian produced the greatest CS improvement. The strength responses of WCP and RCP were also consistent with the corresponding variations in density and UPV.

Jierula et al. [28] found that the WA in FC decreases sharply by up to 45.9% with the increase in density level because of the diminished connectivity of voids and the formation of denser matrix structures. Similarly, Ser et al. [68] found that WA varies within the range of 2.57% to 6.84%, based on the type of aggregate used and the nature of the pore system, emphasizing that while overall porosity plays a critical role in controlling the amount of water absorbed, pore sizes, along with large voids, also play a significant part. In summary, these studies have shown that WA is mainly controlled by different pore structure characteristics, such as overall porosity, pore connectivity and internal density. Indeed, the present study supports this trend, and the WA values significantly decrease in the blends with obsidian powder, with a clear minimum value of 46.9% in S4 compared to the reference sample (S1, 117.7%). The gradual decrease in WA with increasing obsidian replacement is consistent with the concurrent increases in density and UPV, indicating a clear relationship between these physical properties. Conversely, the mixtures with WCP show a non-monotonic behavior and a relatively lower efficiency in WA reduction, suggesting that the influence of WCP on microstructural densification strongly depends on the substitution level without a defined trend. Such non-monotonic behavior is particularly pronounced for S6, where the density decreased to 510 kg/m^3^ and WA increased significantly to 111.1%. The simultaneous decrease in density and increase in WA suggest that density alone does not control the water transport behavior of the WCP mixtures. Pore connectivity and particle packing also play important roles. At the 50% replacement level, the combined particle-size distributions of standard sand and WCP may have produced a less efficient packing arrangement and a more interconnected capillary pore network than those developed at the 25% and 100% replacement levels. This interpretation is also consistent with the relatively lower 7-day and 28-day CS of S6, suggesting reduced matrix compactness. Similarly, the RCP mixtures present overall reduction in WA compared to the reference sample and the improvement in performance is associated with the micro-filler effects and the presence of residual cementitious phases that partially improve the matrix compactness and reduce permeability. Overall, obsidian produced the most consistent reduction in WA, whereas WCP exhibited a non-monotonic response and RCP maintained lower WA values than the reference mixture.

Lin et al. [69] reported that UPV of FC was directly correlated with increasing aggregate replacement ratio and went up from 4557 m/s to 5750 m/s after 28 days (26%), mainly because of decreased porosity and increased continuity of matrix. Roobankumar and SenthilPandian [70] recorded a decline in the UPV when escalating the level of aggregate replacement in polyurethane FC, ranging from 4810 m/s to 3042 m/s (up to 36.75%) due to a higher number of voids and weakening of the internal structure. The study further revealed an excellent relationship between the UPV and CS with respect to the exponential nature of the curve (R^2^ = 0.9012–0.9998). This implies that UPV can be used as a criterion for assessing the internal structural properties of the material. This general trend is also confirmed in the present study where the UPV clearly depends on the type and replacement level of the alternative aggregates. The control specimen (S1) has a value of 1372 m/s. In the mixtures with the addition of the obsidian powder, the UPV increases significantly with increasing substitution, reaching 1506–1795 m/s, indicating an enhanced elastic wave propagation consistent with a more compact and continuous matrix response. This interpretation is also supported by the simultaneous increase in density and CS and the reduction in WA observed in the obsidian series. In contrast, the WCP-containing mixtures show a less consistent UPV response at higher replacement levels, indicating that the effect of WCP on matrix continuity depends on the replacement ratio. Conversely, the RCP mixes show moderately higher UPV values than the reference, which indicates a partial microstructural refinement due to filler effects and residual cementitious activity. In general, the results confirm that UPV is mainly controlled by the microstructural continuity and compactness, with obsidian giving the most significant improvement, RCP a moderate contribution and WCP a limited performance.

Mydin et al. [71] demonstrated that the CS of FC decreases gradually with increasing temperature due to microstructural damage and phase transformation. The initial strength is retained at about 95% up to 200 °C, then reduces to 66.85–75.66% at 400 °C and 36–50.17% at 600 °C, while at 800 °C severe degradation and spalling occurs. This tendency is mainly due to the loss of moisture, coalescence of pores and degradation of cement reaction products such as C-S-H and portlandite leading to the weakening of the matrix. Similarly, Ding et al. [72] reported that CS remains almost unchanged at 100 °C (≈99.5%), then decreases to 92.2% at 200 °C and 69.6% at 400 °C, before dropping sharply to 24.8% at 600 °C and 6.6% at 800 °C, associated with internal vapor pressure, microcrack formation, and ongoing matrix deterioration. In agreement with these observations, the present study also shows a clear temperature-dependent decrease in CS for all the mixtures. The reference sample (S1) gradually lost its load-bearing capacity up to 600 °C and completely collapsed at 800 °C. The obsidian powder-containing S4 mixture showed substantially higher residual strength at all temperature levels and even preserved measurable CS at 800 °C. This indicates that the mixture has higher resistance to thermal degradation, as revealed by its higher residual CS at the studied temperatures. However, strength reduction was also observed in the S7 (WCP) and S10 (RCP) mixtures with increasing temperature, but S7 showed relatively better strength retention up to 600 °C, while S10 showed more pronounced deterioration in the early stage, particularly between 200 and 400 °C, and both mixtures finally lost their load-bearing capacity at 800 °C. It should be noted that S1, S4, S7, and S10 were selected to directly compare the reference mixture with the full-replacement mixture of each alternative aggregate. Therefore, the elevated-temperature tests were intended to evaluate the influence of aggregate type at the maximum replacement level rather than to establish a replacement-ratio-dependent trend.

Zhang et al. [73] showed that the freeze–thaw response of FC is controlled by the evolution of its pore system together with crack development. At a sand–binder ratio of 0.25, a clear improvement in frost resistance was achieved, reflected in a 55.24% rising in peak CS and a 12.87% reduction in CS loss after 80 cycles. This behavior is associated with the production of a uniformly distributed void architecture, which delays the onset of cracking and limits damage progression, while higher sand contents disturb pore stability, facilitate moisture penetration, and consequently accelerate deterioration under repeated freeze–thaw exposure. Liang et al. [74] also showed that CS goes down with more freeze–thaw cycles because of internal stresses caused by freezing. However, after 30 cycles in higher-density mixtures, the strength stayed relatively high (90.7–95.9%). This behavior was attributed to lower air-void volume, lower WA, and thicker pore walls, which increase resistance to frost damage, while higher porosity and pore connectivity accelerate deterioration. Current study results are consistent with the above observations and show that aggregate type plays a decisive role in the freeze–thaw durability of foam concrete. The S1 reference sample showed severe surface deterioration with the complete loss of structural integrity after 16 freeze–thaw cycles, while the S4 mixture with 100% obsidian powder was able to largely preserve its original shape and structural integrity throughout the test, demonstrating its better resistance to freeze–thaw damage. The better performance is in agreement with the lower WA, higher density, and higher CS of S4, which suggest a more compact matrix response under the applied test conditions. Conversely, WCP (S7) and RCP (S10) mixtures exhibited very poor freeze–thaw performance. S7 failed prematurely after 10 freeze–thaw cycles, and S10 failed completely after 16 freeze–thaw cycles. The lower freeze–thaw performance of S7 and S10 is consistent with their higher WA and lower CS in comparison with S4, indicating higher susceptibility to damage accumulation during cyclic freeze–thaw. Nevertheless, the 16-cycle exposure provides a preliminary comparative indication of the relative behavior of the investigated mixtures under the applied conditions but offers only limited evidence for establishing their long-term freeze–thaw durability.

Gao et al. [75] concluded that the TC declined from 0.325 to 0.245 W/(m·K) (about 24.6%) when steel slag was incorporated into alkali-activated FC, which was attributed to the increased microporosity, unreacted particles, and the generation of amorphous calcium aluminosilicate hydrate (C–A–S–H) gel that interrupted continuous heat transfer paths; furthermore, the TC increased from 0.235 to 0.365 W/(m·K) with increasing density (800–1050 kg/m^3^), confirming the significant influence of void architecture and matrix continuity. Likewise, Liu et al. [76] observed the reduction in TC from 0.1713 to 0.1233 W/(m·K) and 0.1228 to 0.1052 W/(m·K) by incorporating glass hollow microspheres, which was ascribed to the interruption of heat transfer pathways and air entrainment. Abbas et al. [77] reported 50% decline in TC and 24% reduction in density for mixtures containing waste plastic aggregates, which proved the effect of microstructural discontinuities in thermal transfer restriction. Kilincarslan et al. [78] reported parameters varying from 0.125 to 0.179 W/(m·K), with the lowest TC corresponding to the lowest density (463 kg/m^3^), Wei et al. [79] demonstrated a gradual decrease in TC with the reduction in density due to the increased porosity, and Gencel et al. [80] showed that recycled aggregates decreased TC through higher porosity. Bodur et al. [81] reported that the TC increased to 0.741 W/(m·K) due to a denser structure. These studies indicate that pore structure, porosity, density, aggregate characteristics, and matrix continuity are the main factors controlling TC. In agreement with this general behavior, the present study shows that TC slightly decreases at lower replacement levels in the obsidian mixtures (0.08606 to 0.08185 W/(m·K)), whereas a pronounced increase is observed at full substitution (0.13574 W/(m·K)). The TC values of all WCP and RCP mixtures remained higher than that of the reference mixture; WCP showed a progressive increase up to 0.15139 W/(m·K), whereas RCP reached a maximum of 0.13563 W/(m·K) at the 50% replacement level before decreasing to 0.12201 W/(m·K) at full replacement. These results indicate that TC is not governed by replacement level alone but by the combined influence of density, aggregate characteristics, and internal matrix structure, consistent with the controlling factors reported in the literature.

Khan et al. [82] reported that the integration of additional cementitious materials improves gelation and microstructural densification of FC. The generation of C-S-H gel that infiltrates the internal voids and reduces the porosity of the microstructure is favored by higher quantities of fly ash and silica fume. Likewise, Mydin et al. [83] showed that the pozzolan-modified mixtures have denser microstructures with the production of secondary C-S-H and C-A-S-H gels, lower porous connectivity, and a compact transition zone, whereas the control mixtures contain loosely packed gel structures and interconnected pores. Rhodia et al. [19] also reported that the decrease in Ca/Si level is positively correlated with the rise in C-S-H formation and microstructural densification. In accordance with the SEM observations, the present study demonstrates that all the mixtures display similar elemental compositions, with Ca, Si, O, C, Al, Fe and Mg confirming the formation of C-S-H-based hydration products. However, the main difference between the mixtures is governed by the differences in the Ca/Si ratio as measured from localized EDS, which are indicative of relative differences in microstructural development rather than absolute bulk chemistry. Within this context, a general trend of decreasing Ca/Si is observed in the modified systems with respect to the reference sample. This points to a change towards a more polymerized C-S-H-type network and a denser binding matrix in the observed regions. The results overall confirm that, while the basic hydration chemistry is similar for all specimens, the mixture composition controls the local microstructural evolution through Ca/Si-related changes in C-S-H organization.

The XRD patterns showed that S1, S4, S7, and S10 generally contained similar crystalline phases, mainly AFt/AFm, portlandite, quartz, and calcite, while differences were observed in their relative diffraction intensities. Liu et al. [84] reported calcium hydroxide and ettringite as major hydration products in foam concrete prepared using anionic and cationic surfactants with mineral powders and reported that the incorporation of small amounts of mineral powders did not produce new hydration products. Al-Shwaiter and Awang [85] reported that palm oil fuel ash used as a sand replacement in foam concrete had a limited effect on the crystalline XRD pattern, while calcite, ettringite, and quartz were also identified in the investigated specimens. The similarity of the identified phase assemblages suggests that the incorporation of the alternative aggregates primarily affected the relative characteristics of the crystalline phases rather than resulting in distinctly different crystalline phase assemblages. To conclude, the XRD results were discussed together with the mechanical, physical and SEM-EDS results in an effort to interpret the effect of the alternative aggregates.

The findings are in line with the current knowledge of void structure–behavior relationships in FC. However, to place the results in the current literature, a wider comparison with recent studies is required. In recent years, studies on FC systems have shown considerable variability in terms of binder type, aggregate type, mixture design and pore-structure-modification strategies. Table 3 presents the mixture compositions and experimental parameters of the FC systems analyzed recently (2025–2026). These studies optimize mechanical, durability, TC and microstructural performance by using various waste-derived aggregates and supplementary materials. Extensive use of waste-derived products and recycled construction materials has been made to improve the matrix compactness and reduce porosity [86,87,88]. Among these, only a limited number of studies have considered WCP and RCP specifically as alternative aggregate types in FC systems. However, the use of obsidian, a geologically formed amorphous volcanic glass with high silica content, is very limited in the extant literature. In particular, there is no clear evidence of a comprehensive assessment of obsidian as an alternative aggregate in FC systems. This study evaluates this limitation by presenting a systematic investigation on the impact of WCP, RCP, and obsidian as alternative aggregates on the engineering properties of the FC.

To address these limitations, this work systematically studied the impact of replacing standard sand with obsidian, WCP, and RCP at various ratios in FC mixtures. Previous studies have mainly focused on conventional aggregates or individual alternative materials, whereas the present study provides a systematic comparative evaluation of three distinct alternative aggregates (obsidian, WCP, and RCP) in FC mixtures. Moreover, a detailed experimental frame work including CS, WA, UPV, elevated temperature exposure and freeze–thaw cycles was adopted to assess the mechanical and durability behaviors. The results show the different impacts of obsidian, WCP and RCP on the mechanical, durability, thermal and microstructural properties of FC. The comprehensive understanding of the behavior of FC with alternative aggregates is achieved by combining the evaluation of these properties and comparing them with previous studies on FC.

## 5. Conclusions

This study presents a systematic assessment of FC performance with obsidian, WCP, and RCP as substitution materials for standard sand at various levels. The microstructure, thermal, durability and mechanical parameters were studied in detail to assess the influence of these alternative aggregates on the matrix system and the overall behavior of the composite. The main conclusions can be summarized as follows:

- The CS increased from 0.545 MPa in the reference mixture to 2.590 MPa at 100% replacement with obsidian as an alternative aggregate. RCP mixtures were consistently greater than 1.0 MPa and WCP mixtures showed moderate gains with a maximum value of 0.824 MPa.
- A reduction in WA was observed with the increasing replacement ratio for the obsidian-based mixtures, with a minimum value of 46.9%. For RCP mixtures, the values mostly remained below the reference mixture of 117.7% in the range of 50.4% to 62.8%. The WCP mixtures showed a variable response in the range of 60.8% to 111.1%, indicating that WA behavior is primarily governed by the effectiveness of pore blocking and capillary network discontinuity induced by the aggregate type and replacement level.
- The UPV results indicate an overall improvement in ultrasonic wave propagation with alternative aggregates compared to the control mixture with a value of 1372 m/s, reaching a maximum value in the obsidian-based system with 1795 m/s, while RCP mixtures also exhibit higher values than the reference up to 1664 m/s, and WCP mixtures show a moderate increase at partial replacement followed by a decrease below the control at full substitution with 1355 m/s, demonstrating that matrix continuity and internal compactness govern ultrasonic transmission performance across all mixtures.
- The TC results demonstrate a clear dependence on both replacement level and alternative aggregate type, where values at low substitution levels remain close to or slightly below the reference mixture of 0.08606 W/(m·K), reaching a minimum of 0.08185 W/(m·K) in the obsidian-based system, while RCP mixtures exhibit intermediate values above the reference, and TC consistently increases across all systems at higher replacement ratios, attaining a maximum of 0.15139 W/(m·K) in the WCP full replacement system, indicating that TC was strongly dependent on the alternative aggregate type and replacement level.
- The values of CS obtained at high temperatures show a continuous decreasing trend under increasing thermal exposure across all tested samples; S1 completely lost its load-bearing capacity at 800 °C, while S4 maintained the highest residual strength of 0.5936 MPa. The CS values obtained at high temperature show a continuous decreasing trend with increasing thermal exposure for all the tested samples. S1 completely lost its load-bearing capacity at 800 °C while S4 showed the highest residual strength of 0.5936 MPa. S7 presented a gradual decrease up to 600 °C and failure at 800 °C, and S10 showed a sharper initial reduction at 400 °C, with a subsequent more gradual decline up to 600 °C. S7 and S10 also lost strength at 800 °C.
- The freeze–thaw test results show that the reference S1, S7 and S10 specimens completely lost their structural integrity after 16 cycles, with WCP failing prematurely after 10 cycles, while the obsidian-based mixture S4 kept a compact and coherent structure, indicating comparatively better performance under the applied 16-cycle freeze–thaw exposure.
- The SEM–EDS and XRD analyses confirmed that the pore architecture of all mixtures was heterogeneous, consisting of C–S–H gel, local microcracks, unreacted particles and AFt crystals, together with portlandite phases. Ca, Si, O, C, Al, Fe and Mg were detected in all specimens, while the Ca/Si ratios varied from 4.09 in S1 to 2.25 in S7.

The current study provides a detailed evaluation of FC with partial and full substitution of standard sand with obsidian, WCP, and RCP in a unified experimental framework. The outcomes highlight that the combined effects of void-architecture optimization and matrix compaction rather than the replacement of aggregate alone are associated with the observed mechanical and durability performance. In this context, these alternative aggregates are not merely conventional aggregate replacements but materials influencing the continuity of the internal structure, the water absorption behavior, and the strength development. The principal scientific contribution of this study is the direct comparison of the relative effects of obsidian, WCP, and RCP on the mechanical, physical, thermal, and durability performance of FC. From the application perspective, the use of obsidian, WCP, and RCP suggests a feasible route for producing lightweight FC for selected construction applications. The selection of alternative aggregate type and replacement level should therefore consider the observed differences in mechanical, thermal, and durability performance. However, practical implementation should consider the regional availability of obsidian, possible variations in RCP properties associated with material source and production batch, and the less consistent performance of WCP at elevated replacement ratios. The findings of this study may encourage future studies to optimize the mixture parameters and replacement levels to further enhance the performance characteristics. Further, prolonged durability assessment throughout sustained environmental factors like freeze–thaw cycles and high temperature effects would give a deeper understanding of the performance of FC incorporating alternative aggregates. Moreover, advanced pore structure characterization techniques and large-scale application studies can help to understand the long-term performance of such materials.

Future recommendations

Future research should address the performance of FC incorporating obsidian, WCP, and RCP in challenging conditions, including long-term freeze–thaw cycles, elevated temperatures, and aggressive chemical exposure to assess its durability and strength development. Another possible area of future research could be the optimal design of the percentage of replacement and mixture proportions for enhancing the performance and durability of FC with these alternative aggregates. More advanced techniques of pore structure characterization could be implemented to more precisely quantify the relationship between void architecture and material functionality. In a more practical sense, production trials and structural uses of FC made from obsidian, WCP, and RCP need to be reviewed for their feasibility when used in the construction industry. Furthermore, life-cycle assessment of the material would provide valuable insight into the sustainability potential of replacing conventional aggregates with natural and recycled alternative materials.

## Figures and Tables

**Table 1:** Chemical composition of cement, RCP, WCP, and obsidian powder.

Component	Cement	RCP	WCP	Obsidian
Na_2_O	0.5605	3.6760	5.3058	4.0181
MgO	1.6626	3.0802	0.8369	0.3428
Al_2_O_3_	5.5654	12.9391	19.8151	13.8233
SiO_2_	20.8669	53.5557	69.9115	71.5910
P_2_O_5_	0.1235	0.1567	0.1760	0.0631
SO_3_	2.7589	0.6022	0.0177	0.1114
K_2_O	1.2203	0.9458	1.3257	4.9842
CaO	58,9222	11.0889	0.7955	2.7944
TiO_2_	0.4525	0.5331	0.5765	0.1876
Cr_2_O_3_	—	0.0381	0.0457	—
MnO	0.0809	0.1581	—	0.0496
Fe_2_O_3_	3.6490	6.0152	0.8755	1.5196
ZnO	0.2482	—	—	—
Loss on ignition (L.O.I.)	3.8890	7.2110	0.3180	0.5150

**Table 2:** Mixture proportions (g).

Sample ID	Replacement Level (%)	Standard Sand	Obsidian Powder	WCP	RCP	Cement	Water	Foam
S1	0	133.33	0.00	0	0	426.67	186.67	225.9
S2	25	100.00	33.33	0	0	426.67	186.67	225.9
S3	50	66.67	66.67	0	0	426.67	186.67	225.9
S4	100	0.00	133.3	0	0	426.67	186.67	225.9
S5	25	100	0	33.33	0	426.67	186.67	225.9
S6	50	66.67	0	66.67	0	426.67	186.67	225.9
S7	100	0	0	133.33	0	426.67	186.67	225.9
S8	25	100	0	0	33.33	426.67	186.67	225.9
S9	50	66.67	0	0	66.67	426.67	186.67	225.9
S10	100	0	0	0	133.33	426.67	186.67	225.9

**Table 3:** Comparative analysis of FC systems incorporating alternative aggregates in recent studies.

Studies	Year	Material Type	Binder Type	Aggregate Type	Replacement Level (%)	Key Tests	Main Focus
This Study	2026	FC	Portland cement	Obsidian, WCP, RCP	25, 50, 100	CS, UPV, TC, WA, high-temperature, SEM–EDS	Comparative assessment of mechanical, physical, thermal, durability, and microstructural performance
Zhou et al. [20]	2026	FC	Portland cement	Bamboo aggregate (BA)	BA: 1.25–5	CS, TC, acoustic properties, WA, SEM	Development of bio-based FC with enhanced thermal and acoustic performance
Bayraktar et al. [21]	2026	FC	Portland cement + ceramic powder	Coconut shell aggregate (CSA)	CP: 0–20 CSA: 0–100	CS, sorptivity, TC, high-temperature, freeze–thaw	Sustainable aggregate use and durability enhancement
Fan et al. [86]	2025	FC	Portland cement + silica fume (SF), mineral powder (MP), metakaolin (MK)	Expanded perlite aggregate (EP)	SF: 3–9 MP: 3–9 MK: 3–9 EP: 100	CS, TC, WA, shrinkage	Influence of supplementary cementitious materials on mechanical and thermal performance
Yılmazoğlu et al. [87]	2025	Alkali-activated FC	Ground granulated blast furnace slag (GBFS) + fly ash (FA) + clinoptilolite zeolite (CZ)	Pumice aggregate (PA)	FA: 5, 10, 20 CZ: 5, 10, 20 PA: 100	CS, TC, high-temperature, freeze–thaw	Influence of FA and CZ on durability and thermal performance
Mydin et al. [88]	2025	FC	Portland cement	Waste sanitary ware (WSW)	WSW: 5–25% (optimum 10%)	CS, WA, sorptivity, porosity, TC, SEM	Enhancement of pore structure and durability using recycled ceramic aggregate
Thangavel et al. [89]	2026	FC	Portland pozzolana cement + sugarcane bagasse ash (SCBA)	Quarry micro fines (QMF)	QMF: 0–100 SCBA: 0–20	CS, WA, porosity, UPV, MIP	Optimization of mechanical performance and pore structure using waste materials
Hamadi et al. [90]	2026	FC	CEM II/A 42.5 cement	Olive stone aggregate	10	CS, TC, Density, Specific heat	Enhancement of thermal insulation and mechanical performance using organic waste aggregates
Shi et al. [91]	2026	FC	Portland cement	Ceramsite aggregate	20, 30, 35, 50	Dynamic mechanical behavior, energy absorption, SEM	Enhancement of impact resistance and energy absorption capacity
Lyu et al. [92]	2026	FC	Portland cement	Oyster shell aggregate	10, 15, 20	CS, splitting tensile strength, chloride penetration	Development of eco-friendly FC with improved marine durability

## Data Availability

The original contributions presented in this study are included in the article. Further inquiries can be directed to the corresponding author.
